# Local dendritic balance enables learning of efficient representations in networks of spiking neurons

**DOI:** 10.1073/pnas.2021925118

**Published:** 2021-12-07

**Authors:** Fabian A. Mikulasch, Lucas Rudelt, Viola Priesemann

**Affiliations:** ^a^Max Planck Institute for Dynamics and Self-Organization, 37077 Göttingen, Germany;; ^b^Bernstein Center for Computational Neuroscience Göttingen, 37077 Göttingen, Germany

**Keywords:** efficient coding, synaptic plasticity, balanced state, neural sampling, dendritic computation

## Abstract

How can neural networks learn to efficiently represent complex and high-dimensional inputs via local plasticity mechanisms? Classical models of representation learning assume that feedforward weights are learned via pairwise Hebbian-like plasticity. Here, we show that pairwise Hebbian-like plasticity works only under unrealistic requirements on neural dynamics and input statistics. To overcome these limitations, we derive from first principles a learning scheme based on voltage-dependent synaptic plasticity rules. Here, recurrent connections learn to locally balance feedforward input in individual dendritic compartments and thereby can modulate synaptic plasticity to learn efficient representations. We demonstrate in simulations that this learning scheme works robustly even for complex high-dimensional inputs and with inhibitory transmission delays, where Hebbian-like plasticity fails. Our results draw a direct connection between dendritic excitatory–inhibitory balance and voltage-dependent synaptic plasticity as observed in vivo and suggest that both are crucial for representation learning.

## Significance

Neurons have to represent an enormous amount of sensory information. To represent this information efficiently, neurons have to adapt their connections to the sensory inputs. An unresolved problem is how this learning is possible when neurons fire in a correlated way. Yet, these correlations are ubiquitous in neural spiking, either because sensory input shows correlations or because perfect decorrelation of neural spiking through inhibition fails due to physiological transmission delays. We derived from first principles that neurons can, nonetheless, learn efficient representations if inhibition modulates synaptic plasticity in individual dendritic compartments. Our work questions pairwise Hebbian plasticity as a paradigm for representation learning and draws a link between representation learning and a dendritic balance of input currents.

Many neural systems have to encode high-dimensional and complex input signals in their activity. It has long been hypothesized that these encodings are highly efficient; that is, neural activity faithfully represents the input while also obeying energy and information constraints (1–3). For populations of spiking neurons, such an efficient code requires two central features: First, neural activity in the population has to be coordinated, such that no spike is fired superfluously (4); second, individual neural activity should represent reoccurring patterns in the input signal, which reflect the statistics of the sensory stimuli (2, 3). How can this coordination and these efficient representations emerge through local plasticity rules?

To coordinate neural spiking, choosing the right recurrent connections between coding neurons is crucial. In particular, recurrent connections have to ensure that neurons do not spike redundantly to encode the same input. An early result was that in unstructured networks the redundancy of spiking is minimized when excitatory and inhibitory currents cancel on average in the network (5–7), which is also termed loose global excitatory–inhibitory (E-I) balance (8). To reach this state, recurrent connections can be chosen randomly with the correct average magnitude, leading to asynchronous and irregular neural activity (5) as observed in vivo (4, 9). More recently, it became clear that recurrent connections can ensure a much more efficient encoding when E-I currents cancel not only on average, but also on fast timescales and in individual neurons (4), which is also termed tight detailed E-I balance (8). Here, recurrent connections have to be finely tuned to ensure that the network response to inputs is precisely distributed over the population. To achieve this intricate recurrent connectivity, different local plasticity rules have been proposed, which robustly converge to a tight balance and thereby ensure that networks spike efficiently in response to input signals (10, 11).

To efficiently encode high-dimensional input signals, it is additionally important that neural representations are adapted to the statistics of the input. Often, high-dimensional signals contain redundancies in the form of reoccurring spatiotemporal patterns, and coding neurons can reduce activity by representing these patterns in their activity. For example, in an efficient code of natural images, the activity of neurons should represent the presence of edges, which are ubiquitous in these images (3). Early studies of recurrent networks showed that such efficient representations can be found through Hebbian-like learning of feedforward weights (12, 13). With Hebbian learning the repeated occurrence of patterns in the input is associated with postsynaptic activity, causing neurons to become detectors of these patterns. Recurrent connections indirectly guide this learning process by forcing neurons to fire for distinct patterns in the input. Recent efforts rigorously formalized this idea for models of spiking neurons in balanced networks (11) and spiking neuron sampling from generative models (14–17). The great strength of these approaches is that the learning rules can be derived from first principles and turn out to be similar to spike-timing–dependent plasticity (STDP) curves that have been measured experimentally.

However, to enable the learning of efficient representations, these models have strict requirements on network dynamics. Most crucially, recurrent inhibition has to ensure that neural responses are sufficiently decorrelated. In the neural sampling approaches, learning therefore relies on strong winner-take-all dynamics (14–17). In the framework of balanced networks, transmission of inhibition has to be nearly instantaneous to ensure strong decorrelation (18). These requirements are likely not met in realistic situations, where neural activity often shows positive correlations (19–22).

We here derive a learning scheme that overcomes these limitations. First, we show that when neural activity is correlated, learning of feedforward connections has to incorporate nonlocal information about the activity of other neurons. Second, we show that recurrent connections can provide this nonlocal information by learning to locally balance specific feedforward inputs on the dendrites. In simulations of spiking neural networks we demonstrate the benefits of learning with dendritic balance over Hebbian-like learning for the efficient encoding of high-dimensional signals. Hence, we extend the idea that tightly balancing inhibition provides information about the population code locally and show that it can be used not only to distribute neural responses over a population, but also for an improved learning of feedforward weights.

## Results

The goal in this paper is to efficiently encode a continuous high-dimensional input signal by neural spiking. In the following, we explain how neurons can learn efficient representations of these inputs through local plasticity mechanisms. We first show how a tight somatic balance can guide neural spiking to distribute the encoding over the population. We then show how a tight balance on the level of dendrites can guide the learning of efficient representations in the feedforward weights.

### Background: Efficient Encoding by Spiking Neurons with Tight E-I Balance

#### Setup

**Fig. 1.**
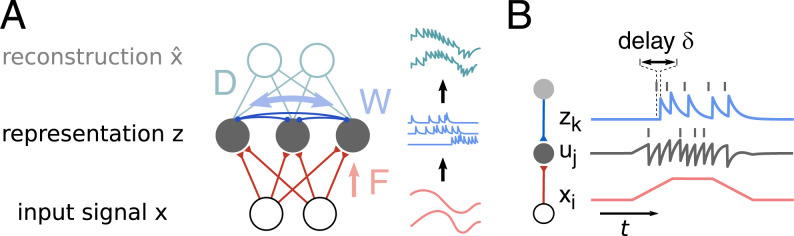
The task is to efficiently encode analog input signals **x** by the response of a population of spiking neurons **z**. (*A*) To that end, neurons couple to the input via feedforward weights *F* (dominated by excitation) and to each other via recurrent weights *W* (dominated by inhibition). From the encoding an external observer can decode an approximation $\hat{x}$ of the original input signal **x** via a linear transformation *D*. (*B*) The membrane potential *u_j_* of neuron *j* is a linear sum of continuous inputs *x_i_* and spike traces *z_k_*. Spikes cause an immediate self-inhibition, which can be seen as an approximate reset of *u_j_*. Spikes of other neurons are transmitted with a delay *δ*. When recurrent weights are learned such that recurrent input *z_k_* cancels feedforward input *x_i_*, *u_j_* is balanced and reflects the global encoding error $x-\hat{x}$. In that case, spikes are fired only when the encoding error is high, so that the spike encoding is efficiently distributed over the population.

Continuous spatiotemporal inputs x(t) drive a recurrently connected spiking neural network, which encodes the inputs through responses z(t) (Fig. 1*A*). Feedforward weights *F_ji_* indicate how strongly inputs $x_{i}(t)$ couple to neuron *j*, and recurrent weights *W_jk_* provide coupling between the neurons. Inputs $x_{i}(t)$ are always positive, to ensure that single synapses act either excitatory or inhibitory, but not both. Neurons in the network encode the inputs by emitting spikes, which then elicit postsynaptic potentials (PSPs) z(t). The PSPs are modeled as a sum of exponentially decaying depolarizations $z_{j}(t)=\sum_{t_{s}^{j}\leq t-\delta }\exp\;(-\frac{t-\delta -t_{s}^{j}}{\tau })$ with decay time *τ* for each spike of neuron *j* at times $t_{s}^{j}$. PSPs arrive after one timestep *δ*, which we interpret as a finite transmission delay of neural communication. Our model is similar to those in previous studies of balanced spiking networks (11, 23).

The goal is to find the most efficient spike encoding that enables the best reconstruction of the input, while the average firing rate of individual neurons is held fixed (see *SI Appendix*, section B for details). To test the reconstruction of the input, we consider the best linear readout $\hat{x}(t)=Dz(t)$ from the neural response and quantify the mean decoder loss

[1] $$\begin{array}{l} L=\frac{1}{2N_{x}}{\langle ||x(t)-\hat{x}(t)|{|}^{2}\rangle }_{t}=\frac{1}{2N_{x}}{\langle ||x(t)-Dz(t)|{|}^{2}\rangle }_{t} \end{array},$$

where *N_x_* is the number of inputs. It is important to note that the readout is not part of the network, but serves only as a guidance to define a computational goal. Hence, learning an efficient code amounts to minimizing L via local plasticity rules on *F_ji_* and *W_jk_*, given the best decoder *D* and a fixed firing rate.

#### Spiking neuron model

Spiking neurons are modeled as stochastic leaky integrate-and-fire (LIF) neurons. More precisely, the model employed here is a special case of the spike response model with escape noise, which is a phenomenological noise model that summarizes effects of biophysical channel noise as well as stochastic input on neural spiking (24). This stochasticity of spiking is required, since deterministic neurons in balanced networks with transmission delays lead to erratic network behavior (18), and it allows a direct link to neural sampling and unsupervised learning via expectation–maximization (*SI Appendix*, section B). A neuron *j* emits spikes with a probability that depends on its membrane potential $u_{j}(t)$ according to

**Fig. 2.**
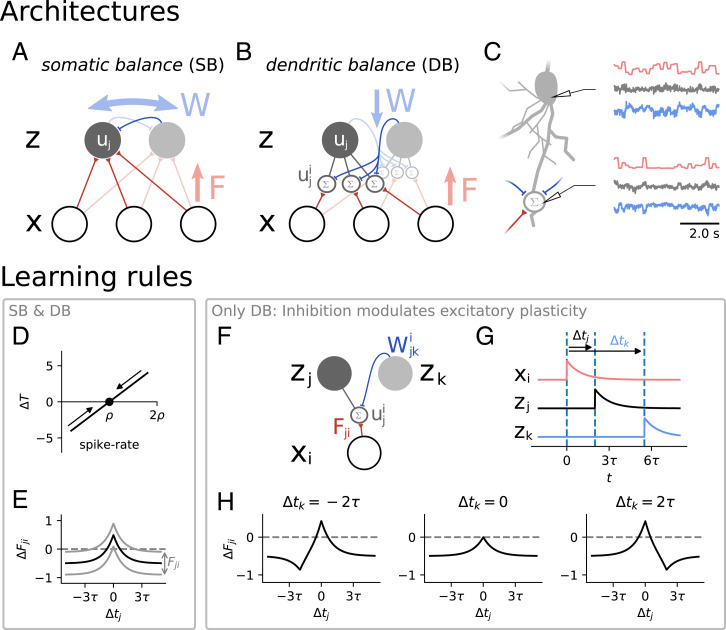
We compare learning in two network models, a point neuron model with somatic balance, and a model with dendritic balance. (*A*) In the model with SB, neurons (gray circles) with outputs **z** receive feedforward network inputs **x** (white circles) and are coupled via recurrent connections. Recurrent weights *W* are adapted to balance other inputs to the somatic membrane potential *u_j_*, which ensures an efficient spike encoding. (*B*) In our proposed model with DB, neurons receive inputs at specific dendritic compartments. Recurrent connections learn to balance input currents locally at the dendrites. This leads to dendritic potentials $u_{j}^{i}$ that are proportional to the coding error for specific feedforward inputs and therefore can be used to learn feedforward weights. (*C*) After learning, local feedforward (red) and recurrent (blue) currents have adapted to tightly balance each other in individual dendritic compartments (*Bottom*). This dendritic balance also results in a somatic balance of inputs (*Top*). Here we show a neuron from a network with 80 neurons coding for natural images. (*D*) In both models a rapid compensatory mechanism ensures that every neuron fires with rate *ρ*. If any neuron spikes too rarely, its threshold *T_j_* is lowered; if it spikes too often, *T_j_* is increased. (*E–H*) Illustration of learning rules in terms of experimental STDP rules. For easier interpretability we plot weight changes for spiking inputs *x_i_*, whereas in the remainder of this paper, *x_i_* are analog input signals. (*E*) For learning feedforward weights in the point neuron model (SB) a Hebbian-like STDP rule increases or decreases weights *F_ji_* depending on the time difference between pre- and postsynaptic spikes $\Delta t_{j}$ and the weight *F_ji_* itself. If *F_ji_* is high or low, this shifts plasticity toward depression or potentiation, respectively. The same learning rule applies to the DB model, if a neuron does not simultaneously receive any recurrent input. (*F–H*) Illustration of how inhibition modulates feedforward plasticity in the proposed model for a network of two coding neurons *z_j_* (with one dendritic compartment) and *z_k_* and one input neuron *x_i_*. (*F*) The excitatory weight *F_ij_* and the inhibitory weight $W_{jk}^{i}$ attach to the same dendritic potential $u_{j}^{i}$. (*G*) We consider the following example where three spikes are fired: *x_i_* at *t* = 0, *z_j_* at $t=\Delta t_{j}$, and *z_k_* at $t=\Delta t_{k}$. (*H*) The total change in the weight *F_ji_* depends not only on the spike time difference $\Delta t_{j}$ between the input and the postsynaptic neuron, but also on the relative inhibitory spike time $\Delta t_{k}$. In general, if *z_j_* and *z_k_* spike close together, *F_ji_* will tend to be depressed. All weight changes were calculated with $F_{ji}=-W_{jk}^{i}=0.5$.

[2] $$p_{\text{spike}}(u_{j}(t))=\text{sig}\left(\frac{u_{j}(t)-T_{j}}{\Delta u}\right),$$

where $\text{sig}(x)={\left[1+\exp\;(-x)\right]}^{-1}$ is a sigmoid function. When the membrane potential approaches the firing threshold *T_j_*, the firing probability increases rapidly. To fix the number of spikes for an efficient code, *T_j_* is adapted to control the average firing rate of each neuron (Fig. 2*C*). Furthermore, Δu regulates the stochasticity of spiking. For increasing Δu the spike emission becomes increasingly noisy, whereas for Δu→0 one recovers the standard LIF neuron with sharp threshold. The membrane potential itself is modeled as a linear sum of the feedforward inputs $x_{i}(t)$ and recurrent inputs $z_{k}(t)$; i.e.,

[3] $$u_{j}(t)=\underset{\text{feedforward}\;\text{input}}{\underset{︸}{\sum_{i}F_{ji}x_{i}(t)}}+\underset{\text{recurrentinput}}{\underset{︸}{\sum_{k}W_{jk}z_{k}(t)}}.$$

Note that, for simplicity, in this model coding neurons are directly coupled by inhibitory connections, but similar dynamics and learning behavior can be implemented in networks with inhibitory interneurons (11).

#### Learning an efficient spike encoding with recurrent plasticity.

Spiking neurons can efficiently distribute neural responses to the input signals over the population, by tightly balancing feedforward and recurrent input at the soma (4, 11) (Fig. 1*B*). In fact, a tight balance of inputs is a direct consequence of learning an efficient encoding via gradient descent on the decoder loss (see *SI Appendix*, section B for derivation). To learn a tight balance recurrent weights adapt according to

[4] $$\Delta W_{jk}\propto -z_{k}u_{j}\;(\text{somatic}\;\text{balance}).$$

Hence, when neuron *k* is active and the somatic potential of neuron *j* is out of balance, i.e., $u_{j}(t)\neq 0$, the weight *W_jk_* changes to balance $u_{j}(t)$. Note that all neurons have an autapse that learns to balance their own membrane potentials, which can alternatively be interpreted as an approximate membrane potential reset after spiking.

This tight balance enables an efficient encoding, since once an input signal is encoded by the spike of a coding neuron, this spike will approximately cancel the excitatory feedforward input to all other neurons and therefore discourage further spiking. More technically, learning a balance with recurrent plasticity leads to recurrent weights that “decode” the population activity onto the membrane potential of each individual neuron $W_{jk}=-\sum_{i}F_{ji}D_{ik}$ (where *D_ik_* is the optimal decoder). The membrane potentials thus reflect the coding error $u_{j}(t)=\sum_{i}F_{ji}\left(x_{i}(t)-{\hat{x}}_{i}(t)\right)$, i.e., the global coding goal, and subsequently drive spiking only when the global encoding is not capturing the signal well.

### Learning Efficient Representations with Feedforward Plasticity

To enable an efficient encoding of high-dimensional signals, feedforward weights *F* should be adapted to the statistics of the input signal. To that end, it is possible to derive a plasticity rule for weights *F_ji_* that minimizes the decoder loss L via gradient descent (*SI Appendix*, section B), which yields

[5] $$\Delta F_{ji}\propto z_{j}(x_{i}-{\hat{x}}_{i})=z_{j}(x_{i}-\sum_{k}D_{ik}z_{k}).$$

Intuitively, this rule drives neuron *j* to correlate its output *z_j_* to input *x_i_*, except if the population is already encoding it. To extract the latter information, the plasticity rule requires a decoding ${\hat{x}}_{i}=\sum_{k}D_{ik}z_{k}$, which contains information about the neural code for input *i* of all other neurons in the population.

We thus conclude that an efficient code relies on information about other neurons in two ways: 1) Neurons need to know what is already encoded to avoid redundancy in spiking (dynamics), and 2) plasticity of feedforward connections requires to know what neurons encode about specific inputs to avoid redundancy in representation (learning). While recurrent weights *W_jk_* for efficient spiking dynamics 1) can be learned locally (Eq. **4**), learning feedforward synapses *F_ji_* correctly 2) is not feasible locally for point neurons, since they lack knowledge about the population code for single inputs *x_i_*.

In the following, we introduce the main result of this paper: Similar to efficient spiking through a tight balance of all feedforward and recurrent inputs at the soma, local learning of efficient representations can be realized by tightly balancing specific feedforward inputs with recurrent input. Physiologically, we argue that this corresponds to spatially separated inputs at different dendritic compartments, where recurrent connections balance the local membrane potential. We contrast this local implementation of the correct gradient of the decoder loss with a common local approximation of the gradient, which is necessary for point neurons with somatic balance only.

#### Somatic balance alone requires an approximation for local learning

Since synapses for point neurons have no access to the population code for single inputs, previous approaches used a local approximation to $\Delta F_{ji}$ where only pre- and postsynaptic currents are taken into account (Fig. 2*E*):

[6] $$\begin{array}{l} \Delta F_{ji}\propto z_{j}(x_{i}-F_{ji}z_{j})\;(\text{Hebbian-like}\;\text{learning}). \end{array}$$

We refer to this learning scheme, consisting of Eqs. **4** and **6**, as somatic balance (SB). A practically identical setup has been proposed in ref. 11. We take this setup as a paradigmatic example of a larger group of Hebbian-like learning rules, which have been used to model representation learning (for a more detailed discussion of related models and learning rules in the literature see *SI Appendix*, section C).

Such Hebbian-like learning rules follow the correct gradient when neurons do not code simultaneously, and thus nonlocal dependencies during learning are not present. This is the case when only a single PSP $z_{j}(t)$ is nonzero at a time, e.g., in winner-take-all circuits with extremely strong inhibition (15), or when the PSP is extremely short (14). The learning rule becomes also approximately exact when neural PSPs z(t) in the encoding are uncorrelated (11, 12). However, these are strong demands on the dynamics of the network, which ultimately limit its coding versatility and are likely not met under realistic conditions.

#### Dendritic balance allows local learning of efficient representations

When neural PSPs z(t) in the population are correlated, learning efficient representations requires that information about the population code is available at the synapses. To this end, we introduce local dendritic potentials $u_{j}^{i}$ at synapses *F_ji_* and couple neurons *k* via dendritic recurrent connections $W_{jk}^{i}$ to these membrane potentials (Fig. 2*B*). The somatic membrane potential is then realized as the linear sum of the local dendritic potentials

[7] $$\begin{array}{ll} u_{j}(t) & =\sum_{i}u_{j}^{i}(t)\\ u_{j}^{i}(t) & =\underset{\text{feedforward}\;\text{input}}{\underset{︸}{F_{ji}x_{i}(t)}}+\underset{\text{recurrent}\;\text{input}}{\underset{︸}{\sum_{k}W_{jk}^{i}z_{k}(t)}}. \end{array}$$

Note that this amounts only to a refactoring of the equation for the somatic membrane potential and does not change the computational power of the neuron. Given such a network with recurrent weights $W_{jk}^{i}$, a SB network with recurrent weights $W_{jk}=\sum_{i}W_{jk}^{i}$ has equivalent dynamics. Hence, any improvement in the neural code in this setup is due to an improvement in the learning of feedforward weights. In the discussion, we address how the compartmentalization in Eq. **7** could be realized in biological neurons and how one can reduce the amount of recurrent dendritic connections $W_{jk}^{i}$ without losing the central benefits of this model.

Introducing dendritic compartments for individual inputs allows us to use the same trick as before: By enforcing a tight E-I balance locally, recurrent connections will try to cancel the input as well as possible. Thereby, recurrent weights $W_{jk}^{i}$ will automatically learn the best possible decoding of the population activity z to the input $F_{ji}x_{i}$. This leads to a local potential that is proportional to the coding error $u_{j}^{i}=F_{ji}(x_{i}-{\hat{x}}_{i})$. In terms of recurrent synaptic plasticity, this is realized by

[8] $$\Delta W_{jk}^{i}\propto -z_{k}u_{j}^{i}\;(\text{dendritic}\;\text{balance}).$$

Thus, the dendritic membrane potential $u_{j}^{i}$ can be used to find the correct gradient $\Delta F_{ji}$ from Eq. **5** locally:

[9] $$\Delta F_{ji}\propto \frac{1}{F_{ji}}z_{j}u_{j}^{i}\;(\text{learning}\;\text{by}\;\text{errors}).$$

We refer to this learning scheme as dendritic balance (DB). As can be seen, the learning rules for feedforward and recurrent weights both rely on the local dendritic potential, which they also influence. This enables recurrent inputs to locally modulate feedforward plasticity. However, this also requires the cooperation of feedforward and recurrent weights during learning. We propose three different implementations that ensure this cooperation, by learning recurrent weights on a faster or on the same timescale as feedforward weights (*SI Appendix*, section B.3). We show that these three approaches yield similar results, which equal the analytical solution (Eq. **5**) in performance (*SI Appendix*, Figs. S2 and S3).

It is possible to integrate the learning rules that depend on membrane potentials over time and obtain learning rules that depend on the relative spike timings of multiple neurons. If we consider only one input neuron and one coding neuron, learning with dendritic balance and somatic balance yields the same spike-timing–dependent plasticity rule. This rule is purely symmetric and strengthens the connection when both neurons fire close in time (Fig. 2*E*). However, if the spike of the excitatory input neuron is accompanied by an inhibitory spike in the coding population, the spike-timing–dependent rule breaks symmetry (Fig. 2*H*). This shows how learning with dendritic balance can take more than pairwise interactions into account to enable the neuron to find its place in the population code.

### Simulation Experiments

To illustrate the differences that arise between the networks using SB and DB during learning, we set up several coding tasks of increasing complexity. Most centrally, we will show that two aspects of realistic neural dynamics make learning especially difficult: 1) correlated occurrences of the patterns that are represented by coding neurons and 2) transmission delays of recurrent inhibition. Both aspects lead to correlations in the activity of coding neurons **z**, which, as we demonstrate, have a detrimental effect on the representations learned by Hebbian-like learning.

#### Learning an efficient encoding with recurrent and feedforward synaptic plasticity

**Fig. 3.**
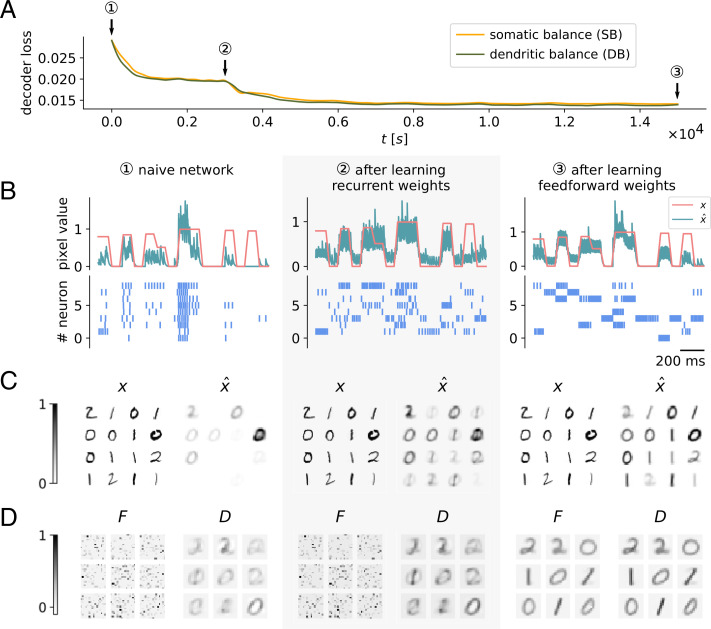
Learning an efficient encoding with recurrent and feedforward synaptic plasticity. In this simulation experiment, networks consisting of nine coding neurons encoded 16 × 16 images of digits 0, 1, and 2 from the MNIST dataset. (*A*) Decoder loss decreases with neural plasticity for both models using either SB or DB. A naive network with random feedforward and zero recurrent weights shows a large decoder loss (*1*). Learning recurrent connections results in a drop in decoder loss (*2*). Later, feedforward plasticity was turned on, also resulting in an improvement of performance (*3*). Final performances and encodings of SB and DB are very similar. (*B–D*) Results of the DB network for different moments in time during learning. (*B*) Input signal *x_i_* and decoded signal ${\hat{x}}_{i}$ for a single pixel *i* in the center of the image. MNIST digits were presented as constant input signals for 70 ms and faded for 30 ms to avoid discontinuities. After learning, the decoded signal tracks the input reasonably well given the very limited capacity of the network. Below are the spike trains of all neurons in the network in response to the input signal. Learning recurrent weights decorrelates neural responses; learning feedforward weights makes neural responses more specific for certain inputs. (*C*) Sample of input images **x** from the MNIST dataset and reconstructions $\hat{x}$ of the input images. The reconstructions presented here are calculated by averaging the decoded signal during input signal presentation over 70 ms. (*D*) Feedforward weights *F* and the optimal decoder *D*. Weights *F* are first initialized randomly; after learning every neuron becomes specific for a certain prototypical digit. Learning also causes feedforward and decoder weights to align.

In a first test we performed a comparison on the MNIST dataset of handwritten digits (Fig. 3*C*). We restricted the dataset to the digits 0, 1, and 2, which were encoded by nine coding neurons. Networks were initialized with random feedforward weights and with zero recurrent weights. To demonstrate the effects of recurrent and feedforward plasticity, we separated learning into two stages: First, recurrent plasticity learned to balance feedforward input to the neurons, which leads to a decorrelation of neural responses to the input signals (Fig. 3*B*), and reduced the decoder loss (Fig. 3*A*). Later, feedforward plasticity was turned on, which aligned feedforward weights with reoccurring patterns in the input (Fig. 3*D*). This further reduced the decoder loss and led to better reconstructions (Fig. 3*C*). Since images were rarely encoded by more than one or two neurons (Fig. 3*B*), interactions in the population were small and thus both setups found similar solutions.

#### Dendritic balance can disentangle complex correlations

**Fig. 4.**
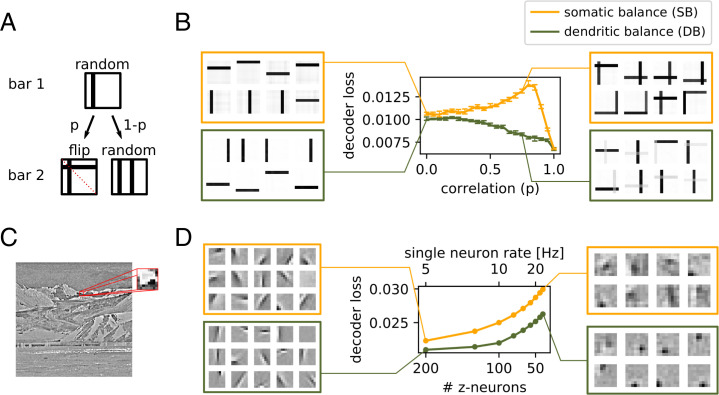
Dendritic balance improves learning for complex correlations in the input signal. (*A* and *B*) In one simulation experiment, 16 neurons code for 8×8 images containing 2 random of 16 possible bars. Thus, optimally, every neuron codes for a single bar. (*A*) Creation of input images with correlation between reoccurring patterns. Two bars are selected in succession and added to the image. With probability *p* the bars are symmetric around the top left to bottom right diagonal axis. With probability 1 –*p* the two bars are chosen randomly. (*B*) Decoder loss after learning for different correlation strengths for networks with SB and DB. Displayed is the median decoder loss for 50 different realizations for each datapoint; error bars denote 95% bootstrap confidence intervals. On the sides, 8 of all 16 converged feedforward weights are shown for representative networks. When correlations between bars are present, the representations learned by SB overlap, while DB still learns efficient single-bar representations. (*C* and *D*) Similarly, for complex natural stimuli DB finds better representations when coding neurons are correlated. (*C*) We extracted 16 × 16-pixel images from a set of whitened pictures of natural scenes (3), scaled them down to 8 × 8 pixels, and applied a nonlinearity (*SI Appendix*, section D). (*D*) Decoder loss after learning of SB and DB networks featuring varying numbers of coding neurons, while keeping the population rate constant at 1,000 Hz. On the sides we show exemplary converged feedforward weights. For a large number of coding neurons (*Left*) both learning schemes yield similar representations, but performance is slightly better for DB. For a small number of neurons (*Right*) DB learns more refined representations with substantially reduced decoder loss compared to SB. The reason is that for a small number of neurons the learned representations are more correlated and consequently are harder to disentangle. Notably, different amounts of neurons result in different coding strategies.

Our theoretical results suggest that DB networks should find a better encoding than SB networks when correlations between learned representations are present in the stimuli. To test this, we devised a variation of Földiak’s bar task (12), which is a classic independent component separation task. In the original task neurons encode images of independently occurring but overlapping vertical and horizontal bars. Since the number of neurons is equal to the number of possible bars in the images, each neuron should learn to represent a single bar to enable a good encoding. We kept this basic setup, but additionally we introduced between-bar correlations for selected pairs of bars (Fig. 4*A*). We then could vary the correlation strength *p* between the bars within the pairs to render them easier or harder to separate.

The simulation results indeed showed that the performance of the SB, but not of the DB model, deteriorates when learned representations are correlated (Fig. 4*B*). The decoder loss for SB grows for increasing *p* and reaches its maximum at about *p* = 0.8. This is because Hebbian-like learning (as used in SB) correlates a neuron’s activity with the appearance of patterns in the input signal, irrespective of the population activity. The correlation between two bars therefore can lead a neuron that initially is coding for only one of the bars to incorporate also the second bar into its receptive field (Fig. 4*B*). Hence, for increasing correlation *p* neurons start to represent two bars, which does not reflect the true statistics of the input, where single bars may still occur. For *p* > 0.8 the decoder loss decreases, as here the occurrence of the correlated pairs of bars becomes so likely that the representations reflect the statistics of the images again. In contrast, DB enables neurons to communicate which part of the input signal they encode and hence they consistently learn to code for single bars. Accordingly, the decoder loss for DB is smaller than for SB for every correlation strength of bars (Fig. 4*B*).

We expected to see a similar difference between SB and DB networks when complex stimuli are to be encoded. In a third experiment we therefore tested the performance of the networks encoding images of natural scenes (Fig. 4*C*). To also test whether the amount of compression (number of inputs vs. number of coding neurons) would affect SB and DB networks differently, we varied the number of coding neurons while keeping the population rate fixed at 1,000 Hz. This way, only the compression, and not also the total number of spikes, has an effect on the performance of the networks.

The simulations showed that for natural images, DB networks learn more efficient representations than SB networks. The difference in performance becomes larger the higher the compression of the input signal by the network is (Fig. 4*D*). This effect seems to be related to the observations we made in the bar task: Networks with few coding neurons have to learn correlated representations (*SI Appendix*, Fig. S10), which renders SB less appropriate. We found that SB networks consistently needed about twice as many neurons to achieve a similar coding performance to that of DB networks (Fig. 4*D*).

#### Dendritic balance can cope with inhibitory transmission delays.

**Fig. 5.**
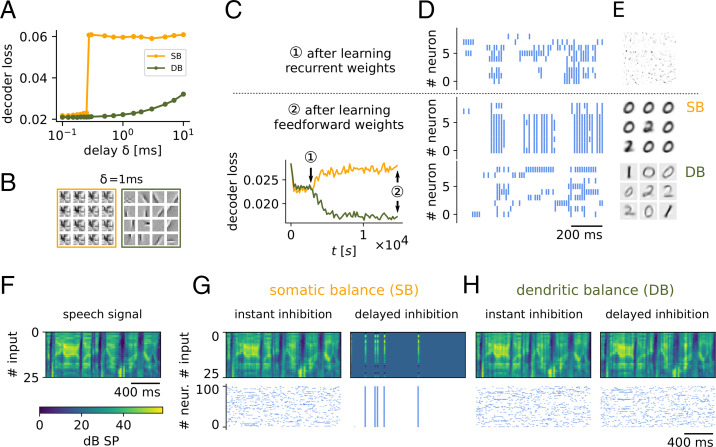
Dendritic balance prevents learning of redundant representations for inhibitory transmission delays. (*A*) Decoder loss of networks of 200 neurons coding for natural scenes for different inhibitory transmission delays *δ*. For transmission delays longer than 0.3 ms, Hebbian-like learning in SB networks leads to highly inefficient representations and large decoder loss. In contrast, for networks learning with DB, the decoder loss increases only moderately even for long transmission delays. The results are robust with respect to the stochasticity of firing Δu and the firing rate *ρ* (*SI Appendix*, Fig. S8). (*B*) Selection of learned weights for a transmission delay of 1 ms. DB learns similar weights as before (Fig. 4*D*), while SB leads to a collapse of representations. (*C–E*) To illustrate the effect of feedforward plasticity, we repeated the MNIST experiment in Fig. 3 with long transmission delays of 3 ms (before, 0.1 ms in Fig. 3). (*C*) First, only recurrent connections were learned (*1*); later, feedforward weights were learned (*2*). As before, recurrent plasticity decorrelates responses and decreases the decoder loss. When feedforward plasticity was turned on, Hebbian-like plasticity (SB) learned worse representations than random feedforward weights, which is indicated by the increase in decoder loss. In contrast, our model with DB learned improved representations with substantially reduced decoder loss. (*D*) The poor performance of the SB model is a consequence of highly synchronous spiking responses to the inputs, whereas neurons fire asynchronously in the model with DB. (*E*) Neurons in the SB model learn overly similar feedforward weights, whereas neurons with dendritic balance learn feedforward weights that capture the input space well. (*F–H*) This effect is still present when input signals show fast changes in time. Here, 100 coding neurons firing at 5 Hz encode a speech signal. (*F*) Spectrogram of the signal presented in 25 frequency channels. (*G*) As can be seen in the reconstructed signal (*Top*), SB finds a good encoding for instant inhibition (loss=0.06), but even for extremely small delays of 0.05 ms the learned representations collapse, leading to pathological network behavior and bad encoding performance (loss=0.23). (*H*) In contrast, DB finds a similar encoding for both instant inhibition (loss=0.057) and inhibitory delays of 0.05 ms (loss=0.06).

Correlations between coding neurons can also be introduced by transmission delays of inhibition (18). We therefore expected to find that DB networks are much more robust to long transmission delays than SB networks. To investigate this, we simulated networks of 200 neurons with a range of timesteps *δ*, which we interpret as transmission delays. We varied the delay from δ=0.1 ms to δ=10 ms and observed how the delay affected coding performance for natural images. Indeed, performance of SB networks drastically broke down to a baseline level when transmission delays became longer than 0.3 ms (Fig. 5*A*). All neurons had learned the same feedforward weights (Fig. 5*B*). In contrast, DB networks continued to perform well even for much longer delays. While long delays for DB also lead to a decrease in coding performance, DB prevented the sudden collapse of the population code.

To illustrate the mechanism that caused the breakdown in performance for SB, we also ran simulations of networks learning to code for MNIST images with longer transmission delays (Fig. 5*C*). After learning with Hebbian-like plasticity, neurons showed highly synchronized activity (Fig. 5*D*) and had learned overly similar feedforward weights (Fig. 5*E*). When transmission delays become long, inhibition will often fail to prevent that multiple neurons with similar feedforward weights spike to encode the same input. Hebbian-like plasticity can exacerbate this effect, since it will adapt feedforward weights of simultaneously spiking neurons in the same direction. In contrast, neurons learning with DB use the information that inhibition provides for learning, even if it arrives too late to prevent simultaneous spiking. Hence DB manages to learn distinct representations also in the face of long transmission delays.

Finally, this difference in the two learning schemes is still present for input signals with fast and complex temporal dynamics. To show this we repeated an experiment from ref. 11, where natural speech sounds were encoded by a population of 100 neurons (Fig. 5 *F*–*H*). In this scenario SB learned only a proper encoding with instantaneous transmission, i.e., when simultaneous spiking was prohibited by removing the least likely spikes in the case of multiple spikes per time bin. However, even for extremely short transmission delays of δ=0.05 ms, Hebbian-like plasticity led to pathological network behavior (Fig. 5*H*). In contrast, DB learned a similarly efficient encoding in both conditions (Fig. 5*G*).

## Discussion

In the past, the formation of neural representations has often been modeled with pairwise Hebbian-like learning rules (11, 12, 14–17, 25–27). However, the learning rules that are derived directly from neural coding models typically require not only information about pre- and postsynaptic activity, but also the coding error of the whole population. Commonly it is maintained that this information is not locally available to the synapse and it is left out of the equation, yielding pairwise Hebbian-like learning rules. Here, we found that omitting this information about the population code can have a detrimental effect on learning when neural activity is correlated, which is the case in realistic conditions. In this case, Hebbian-like learning leads to a highly inefficient encoding in comparison to the derived learning by errors or even in comparison to random connections (Figs. 4 and 5). To overcome this problem, we showed how learning by errors can be implemented locally by neurons with dendritic balance and a voltage-dependent plasticity rule. This suggests that dendritic balance could play a crucial role in synaptic plasticity for the formation of efficient representations.

Why does Hebbian-like learning fail when neural activity is correlated, and how does learning by errors prevent this? When the activity of neurons is correlated, Hebbian-like learning adapts the feedforward weights of these neurons into a similar direction. This even further strengthens the correlations between neurons—a vicious cycle, which ultimately can lead to highly redundant representations and extremely correlated spiking. Strong correlations between coding neurons typically mean that certain inputs are overrepresented and others underrepresented in the population, which is indicated by negative or positive coding errors, respectively. In contrast to Hebbian-like learning, learning by errors selectively weakens connections to overrepresented inputs and thereby helps to reduce the correlations between coding neurons. In our model, correlations between coding neurons can arise through either correlations of the learned representations in the input signal or transmission delays of recurrent inhibition. Correlated firing due to correlations in the input can in principle always be addressed by increasing the number of coding neurons, as this will increase the independence of the learned representations (Fig. 4*D* and *SI Appendix*, Fig. S10). Correlations due to transmission delays of recurrent inhibition, on the other hand, are a fundamental problem that arises in balanced networks (18, 23, 28). Here, the exact point of breakdown of Hebbian learning depends on the specific type of input and network size and might occur for longer transmission delays in simplistic scenarios. However, already in the case of moderately large networks receiving complex input signals the effect is severe—even for submillisecond delays Hebbian-like learning can lead to a collapse of neural representations and almost perfectly correlated spiking of the whole population (Fig. 5). In contrast, learning by errors consistently avoids this breakdown, and we therefore argue that it becomes indispensable when transmission delays are present.

To make coding errors available for single synapses locally, we introduced balanced dendritic potentials that are proportional to these errors. This can be achieved by learning a balance through recurrent plasticity on the dendrites, as then the network automatically finds an optimal decoding of neural activity to the feedforward inputs. Yet, presenting an error through a balance of inputs is a quite general principle, and theoretically it would also be possible to present the coding error elsewhere. Rate-based models of predictive and sparse coding for example suggest that coding errors are presented in the activity of other neural populations (29–32). However, this idea cannot be easily transferred to spiking neurons, where coding errors would be rectified by neural spiking mechanisms; hence, it is not directly possible to present negative and positive errors in the same unit. Neural learning in these theories, however, relies on this, and indeed, still no conclusive experimental evidence for such error units exists (33, 34). Another theory therefore suggests that prediction errors are presented by voltage differences between soma and dendrite in two-compartment neurons (35–37). In contrast, our work shows that a coding error, which is calculated from the mismatch between excitation and inhibition locally in each dendritic compartment, can act as a very precise learning cue for single synapses. What supports this idea is that a local dendritic balance of inputs, which is maintained by plasticity, has indeed been observed experimentally (8, 38–40). Furthermore, this balance on single neurons can also explain central characteristics of cortical dynamics (4), such as highly irregular spiking (41, 42), but correlated membrane potentials of similarly tuned neurons (43, 44).

An apparent downside of implementing dendritic balance is the large increase in the number of recurrent inhibitory connections. Connecting every neuron to each feedforward synapse on the dendrites of other neurons would even for moderately sized networks prove extremely costly. However, we found that only a small fraction of the inhibitory connections in our model are required for learning, namely strong connections between neurons whose firing is correlated. We demonstrated this in the example of the bars task, where 90% of dendritic connections can be pruned without changing the learning outcome (*SI Appendix*, Fig. S9). Moreover, in our model inhibition is mediated by direct recurrent connections between coding neurons, but fewer connections would be required if inhibition was mediated via interneurons. By incorporating inhibitory interneurons with broad feature selectivity, it is possible to merge inhibitory connections that provide largely the same information (11). We therefore expect that the main benefits of the proposed learning scheme can be achieved also with relatively few connections.

### Biological Plausibility

Our model presents the simplest extension of existing point-neuron models (11), which allows us to formally derive and isolate the effect that dendritic balance can have on representation learning. While more complex models of dendritic structure and nonlinear dynamics can elucidate their role for neural computation (45), nonlinear dynamics would also alter the computational capacity (45), thus hampering a direct comparison to previous models of learning. Nevertheless, the question remains whether the proposed learning based on dendritic balance can be implemented by biological neurons. In the following we discuss the main requirements of the proposed learning scheme.

A central element of the dendritic balance model is the dependence of synaptic plasticity on local membrane potentials. Indeed, it has been argued that the local membrane potential is a critical factor determining synaptic plasticity (46–49). Such voltage-dependent plasticity is thought to be mediated mainly by the local calcium concentration, which closely follows the local membrane potential (50, 51) and locally modulates neural plasticity (52). As required by our model, this voltage dependence implies that inhibition can have a large impact on excitatory synaptic plasticity locally (53, 54), which also has been found experimentally (8, 55). Yet, it remains a major open question what the precise functional role of these voltage-dependent plasticity mechanisms could be (8, 45). Our work proposes that a central feature of voltage-dependent synaptic plasticity is to base the plasticity of single synapses not only on pre- and postsynaptic activity, but also on the activity of other neurons in the population.

**Fig. 6.**
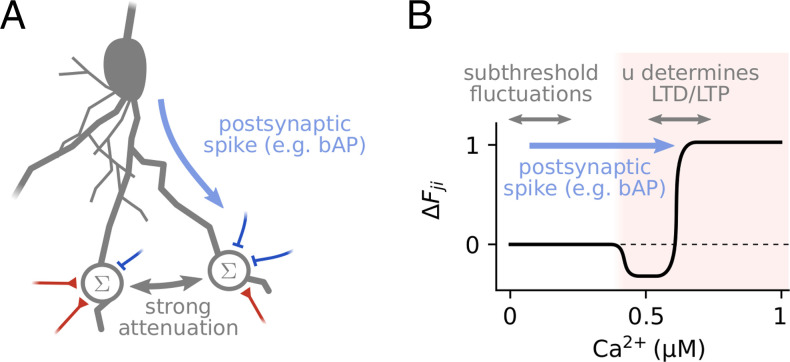
Biologically feasible implementation of the proposed feedforward learning rule. (*A*) The proposed learning scheme requires the following distribution of information in the dendritic tree: First, synapses need to know when a postsynaptic spike occurred. This information could be provided, e.g., by backpropagating action potentials (bAPs). Second, the potentials of the dendritic compartments that sum specific excitatory and inhibitory inputs have to be sufficiently decoupled. Such a strong attenuation of inputs exists for example between dendritic branches (38). (*B*) The inputs $u_{j}^{i}$ to the local potential and the postsynaptic spike signal *z_j_* can be used by a voltage-dependent plasticity rule to implement the proposed learning scheme. Typically such rules assume that plasticity happens in a strongly depolarized regime that is associated with large calcium concentrations (shaded red area, compare to ref. 57). To reconcile our model with such rules, we assume the postsynaptic spike *z_j_* shifts the local potential into the strongly depolarized regime, e.g., through bAPs or dendritic plateau potentials (72), and local input $u_{j}^{i}$ determines whether long-term depression (LTD) or long-term potentiation (LTP) occurs.

How are the proposed learning rules related to experimentally observed voltage-dependent plasticity? Many experiments show that excitatory plasticity requires a strong depolarization of the membrane potential, which for example happens during postsynaptic spiking (46). Our feedforward plasticity rule can be reconciled with plasticity rules that are inspired by these experiments (56, 57) (see Fig. 6*B* for details). The voltage dependence of inhibitory (recurrent) plasticity has only recently started being investigated (8). Recent experimental evidence suggests that this inhibitory plasticity, like excitatory plasticity, is calcium dependent and also requires postsynaptic spiking (58, 59). Our recurrent plasticity rule is similar to previous models of voltage-dependent inhibitory plasticity (11, 60), which set a target value for the postsynaptic membrane potential. Like our rule, these rules have not considered the requirement of postsynaptic spiking for plasticity induction explicitly. We speculate that such a requirement enables the network to preferentially select connections between neurons with correlated activity, which are especially relevant for learning (*SI Appendix*, section B.3). Further experimental and theoretical research is required to understand the precise mechanism and purpose of this type of inhibitory plasticity.

Another requirement of the learning scheme is that different compartments on the dendritic tree are well isolated, so that recurrent inputs can modulate the plasticity of specific synapses (Fig. 6*A*). In biological neurons, dendrites are electronically distributed elements, where strong voltage gradients may exist across the dendritic tree (61, 62). These voltage differences are the result of strong attenuation of input currents, meaning that individual synapses can have very localized effects (63). Thus, the required isolation between compartments exists in biological neurons if they are sufficiently separated and especially for compartments on different dendritic branches (38). This isolation between spatially separated compartments also reduces nonlinear interactions between them. As a result, the integration of any net excitation from different compartments at the soma is approximately linear (63), as required by our model. In contrast to excitation, though, inhibition on distant dendrites mainly acts locally by gating excitation, so that dendritic inhibition can have a very weak effect on the somatic membrane potential (64). Propagating dendritic inhibition to the soma is, however, not required for network function, because any remaining net excitation can also be balanced by plastic inhibitory synapses close to the soma. Therefore, the model’s key requirements for learning and network function could also be met in biological neurons.

However, how synapses in biological neurons are organized on these dendritic compartments seems to be at odds with our model: First, while in our model individual feedforward inputs (which are mostly excitatory) have isolated dendritic potentials, it is well known that correlated excitatory synapses often cluster on dendrites (65–67); second, while in our model we generally find more inhibitory than excitatory synapses, excitatory synapses outnumber inhibitory synapses on dendritic branches, e.g., 4:1 on the dendrites of cultured rat hippocampal neurons (38). We argue that these two disparities can be resolved, if the individual continuous inputs provided to our model are seen as the resulting currents of clustered, correlated synapses. How this clustering could be organized by synaptic plasticity is a matter of ongoing research (68), and it will have to be the subject of future work to reconcile these plasticity mechanisms with representation learning.

### Experimental Predictions

Ultimately, we can generate two directly measurable experimental predictions from our model: First, if input currents to a neuron’s dendrites are locally unbalanced, recurrent plasticity will learn to establish a local E-I balance. Second, our model predicts that the strength of local inhibition determines the sign of synaptic plasticity: During plasticity induction at excitatory feedforward synapses, activating inhibitory neurons that target the same dendritic loci should lead to long-term depression of the excitatory synapses. We would expect this effect to persist, even if the inhibitory signal arrives shortly after the pre- and postsynaptic spiking. These predictions mainly apply to populations of sensory coding neurons, but models similar to the somatic balance model have been proposed to solve other tasks as well (69, 70), suggesting that dendritic balance could be of more general relevance for learning. Indeed, indications of inhibitory modulated plasticity can be found not only in visual cortex (55), but also in hippocampus (38) and possibly other areas (8, 71).

To conclude, we here presented a learning scheme that facilitates highly cooperative population codes for complex stimuli in neural populations. Our results question pairwise Hebbian learning as a paradigm for representation learning and suggest that there exists a direct connection between dendritic balance and synaptic plasticity.

## Materials and Methods

Neural activity was simulated in discrete timesteps of length *δ*. Images were presented as continuous inputs for 100 ms each, that is, as constant inputs for 70 ms, after which they were linearly interpolated over 30 ms to the next image to avoid discontinuities in the input signal. In the speech task, audio signals were encoded in 25 frequency channels, sampled at 200 Hz, and presented with linear interpolation between datapoints. For every experiment a learning set and a test set were created. The networks learned online on the training set; in regular intervals the learning rules were turned off and the performance was evaluated on the test set. Performance was measured via the instantaneous decoder loss (Eq. **1**) by learning the decoder *D* alongside the network. The respective update rule for the decoder is given by

[10] $$\Delta D_{ij}\propto z_{j}(x_{i}-\sum_{k}D_{ik}z_{k}).$$

For DB networks we propose three learning schemes with fast or slow recurrent plasticity (detailed in *SI Appendix*, section B.3). To reduce computation time for large networks, the analytical solution of optimal recurrent weights $W_{jk}^{i}=-F_{ij}F_{ik}$ was used as an approximation of the proposed learning schemes. For Figs. 3 and 5 *C*–*E* the dendritic balance learning scheme with fast recurrent plasticity and the weight decay trick (DB decay in *SI Appendix*) is displayed. For Fig. 4, as well as Fig. 5 *F*–*H*, we used the analytical solution. When comparing the proposed learning schemes to the analytical solution on reference simulations (*SI Appendix*, Figs. S2 and S3), they consistently found very similar network parameters and reached the same performance.

In early simulations we observed that coding performance is largely affected by the population rate, i.e., how many spikes can be used to encode the input signal. To avoid this effect when comparing the two learning schemes, we additionally introduced a rapid compensatory mechanism to fix the firing rates, which is realized by changing the thresholds *T_j_*. We emphasize again that this adaptation is in principle not necessary to ensure stable network function. In fact, error-correcting balanced state inhibition can already be sufficient for a network to develop into a slow firing regime (11). The fixed firing rate is enforced by adapting the threshold *T_j_* according to

$$\Delta T_{j}\propto (s_{j}-\rho \;\delta ),$$

such that neurons are firing with a target firing rate *ρ*. Here, ρ δ is the mean number of spikes in a time window of size *δ* if a neuron would spike with rate *ρ*, and *s_j_* is a spike indicator that is 1 if neuron *j* spiked in the last time *δ*; otherwise *s_j_* = 0.

Furthermore, in the simulations of correlated bars and natural scenes (Fig. 4), we aided the learning process by starting with a high stochasticity in spiking and slowly decreasing it toward the desired stochasticity. While similar results were obtained without using this method, we observed that convergence of the networks to an efficient solution was more reliable with it, as it helped in avoiding local minima of the goal function in early phases of learning. Specifically, we started with a stochasticity of Δu=1.0. We then exponentially annealed it toward the final value $\Delta u^{*}$ by applying every timestep

$$\Delta u(t+1)=\Delta u(t)-\eta_{\Delta u}(\Delta u(t)-\Delta u^{*}).$$

## Supplementary Material

Supplementary File

## Data Availability

Full derivations of the network dynamics and learning rules, more details about the relation of our model to previous models in the literature, and supplementary figures containing additional information for simulation experiments, as well as simulation parameters, are provided in *SI Appendix*. Code for reproducing the main simulations is available in GitHub at https://github.com/Priesemann-Group/dendritic_balance (73). Computer programs data have been deposited in Zenodo at https://zenodo.org/record/4133446.
