# Supplementary material for: Local dendritic balance enables learning of efficient representations in networks of spiking neurons
Source: Proc Natl Acad Sci U S A. 2021 Dec 7;118(50):e2021925118. doi: 10.1073/pnas.2021925118 (PMC8685685; doi:10.1073/pnas.2021925118)
Supplement: Supplementary File [file pnas.2021925118.sapp.pdf]

PNAS
www.pnas.org

Supplementary Information for

Local dendritic balance enables learning of efficient representations in networks of spiking neurons

Fabian A. Mikulasch, Lucas Rudelt, Viola Priesemann

Viola Priesemann.
E-mail: viola.priesemann@ds.mpg.de

This PDF file includes:

Supplementary text
Figs. S1 to S11 (not allowed for Brief Reports)
Table S1 (not allowed for Brief Reports)
SI References

Supporting Information Text

Symbols.

- $\mathbf{X}_{0,T} = \{\mathbf{x}(t)|t \in \{0, ..., T\}\}$: Input signal over time to be encoded
- $\mathbf{S}_{0,T} = \{\mathbf{s}(t)|t \in \{0, ..., T\}\}$: Spikes of coding neurons
- $\mathbf{z}(t)$: 'Outputs' of coding neurons, proportional to evoked post-synaptic potentials
- $\hat{\mathbf{x}}(t) = D\mathbf{z}(t)$: Input signal reconstructed from network activity
- D: Decoder matrix of the decoder model
- σ: Variance of the decoder model
- $\mathbf{b}$: Spiking probability prior of decoder model
- θ: Decoder model parameters $\{D, \sigma, \mathbf{b}\}$
- F: Feedforward weights (mostly excitatory)
- W: Recurrent weights connecting to the soma (mostly inhibitory)
- W^i: Recurrent weights, connecting to the dendrites to input i (mostly inhibitory)
- T_j: Soft threshold of neuron j
- Δu: Stochasticity of neural spiking
- τ: Membrane time constant of leak
- $\eta_{(\cdot)}$: learning rate of parameter $(\cdot)$
- ρ: Target firing rate of neurons
- $1/Z(\cdot)$: Normalization of probability function

A. Stochastic neural dynamics

We simulated stochastic leaky integrate and fire neurons in discrete timesteps. The model can be seen as a special case of the spike response model with escape noise (1). In timestep $t \in \{0, 1, ..., T\}$ with length δ neuron j spikes with a probability

$$p_{\text{dyn}}\left(s_j(t) = 1|\mathbf{x}(t), \mathbf{z}(t)\right) = p_{spike}(u_j(t)) = \text{sig}\left(\frac{u_j(t) - T_j}{\Delta u}\right), \quad [1]$$

where $\text{sig}(x) = [1 + \exp(-x)]^{-1}$, $u_j(t)$ is the membrane potential of the neuron, T_j the firing threshold, Δu defines how stochastic the spiking is and $s_j(t)$ is a spike indicator, which is 1 if neuron j spiked in time step t, otherwise $s_j = 0$. Emitted spikes are then transmitted to other neurons and elicit post synaptic potentials (PSPs) $\mathbf{z}(t)$ with

$$z_j(t) = \sum_{t_s^j < t} \exp\left(-\frac{t - 1 - t_s^j}{\tau}\right),$$

which account for the leaky integration at the membrane. Here, t_s^j are the spike times of neuron j and τ the membrane time constant, which was chosen the same for all neurons. Please note that, in order to ease the upcoming derivations, we changed notation such that t is the index of the discrete timestep and τ has the unit of timesteps. The time delay of PSP arrival of the length of one time step δ is interpreted as a finite traveling time of neural impulses over axons. The PSPs together with input signal $\mathbf{x}(t)$ are then summed up linearly at the soma to give the membrane potential

$$u_j(t) = \sum_i F_{ji} x_i(t) + \sum_k W_{jk} z_k(t).$$

In order to model neurons that make use of dendritic balance we subdivided the somatic potentials such that they are sums of dendritic potentials: $u_j(t) = \sum_i u_i^j(t)$, where the dendritic potentials $u_i^j(t) = F_{ji} x_i(t) + \sum_k W_{jk}^i z_k(t)$. To summarize, stochastic neural dynamics are modeled through the spike probability $p_{\text{dyn}}\left(\mathbf{s}(t)|\mathbf{x}(t), \mathbf{z}(t)\right)$ with neural parameters $\{F, W, \mathbf{T}, \Delta u\}$.

B. Learning an efficient code with expectation maximization

With the following derivations we provide a link between learned balanced state inhibition (2) and neural sampling in graphical models (3). Hence we provide new derivations for the network dynamics and learning rules used in (2), showing how they implement unsupervised learning in a graphical model. Furthermore, with the dendritic balance learning scheme we will address the linear case of the quite general problem that arises through explaining away effects, i.e. converging arrows in graphical models: Converging arrows imply that neurons should cooperate to encode the input and lead to non-localities in update rules when the neural dynamics are based on point neurons. In related studies this problem so far has been avoided in various ways, which all prevent the network from explaining the input through possibly correlated neurons simultaneously and thus limit coding versatility (3–7).

The goal of neural spiking dynamics and plasticity throughout this paper is to find an efficient spike encoding, i.e. representing an input signal $\mathbf{X}_{0,T} = \{\mathbf{x}(t)|t \in \{0, ..., T\}\}$ through a collection of spikes $\mathbf{S}_{0,T} = \{\mathbf{s}(t)|t \in \{0, ..., T\}\}$. $\mathbf{X}_{0,T}$ can be seen here as an episode in an organisms life, which we will assume to be distributed according to $p^*(\mathbf{X}_{0,T})$. We say that $\mathbf{S}_{0,T}$ efficiently encodes $\mathbf{X}_{0,T}$ if the following two conditions are met:

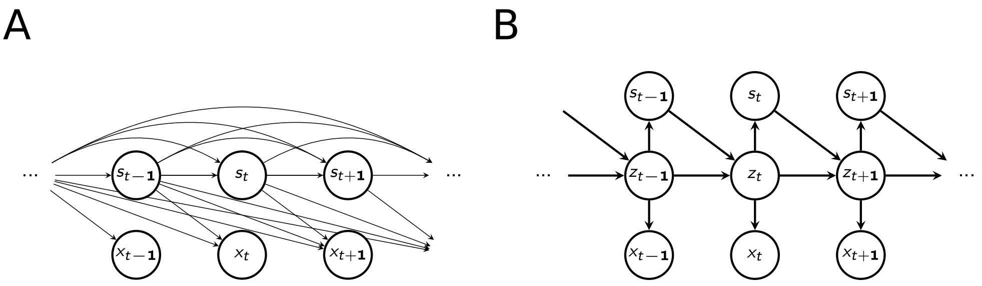

Fig. S1. Graphical representation of the decoder model. **A** We consider a decoding model where readouts of inputs $\mathbf{x}(t)$ (denoted here as $\mathbf{x}_t$) are conditioned on preceding spikes $\mathbf{s}(t)$ (denoted as $\mathbf{s}_t$). **B** By introducing the spike traces $\mathbf{z}(t)$ into the model, the model factorizes over timesteps, which is equivalent to viewing it as a hidden Markov model (HMM) with hidden states $\{\mathbf{z}(t), \mathbf{s}(t)\}$.

a) $\mathbf{X}_{0,T}$ can be accurately estimated from $\mathbf{S}_{0,T}$ via a decoding model $p_\theta(\mathbf{X}_{0,T}|\mathbf{S}_{0,T})$.

b) The number of spikes emitted is small.

Hence we want to maximize the likelihood $p_\theta(\mathbf{X}_{0,T}|\mathbf{S}_{0,T})$ over both the decoding model parameters θ and the latent variables $\mathbf{S}_{0,T}$ sampled by the (constrained) network dynamics $p_{\text{dyn}}(\mathbf{s}(t)|\mathbf{x}(t), \mathbf{z}(t))$.

To show how a stochastic spiking neural network can unsupervisedly learn such an encoding, we make use of the framework of online expectation-maximization (EM) learning (8). EM-learning can find maximum-likelihood estimates for parameters of latent variable models (here $p_\theta(\mathbf{X}_{0,T}, \mathbf{S}_{0,T})$) for observed data ($\mathbf{X}_{0,T}$). For these models it typically is intractable to marginalize out the latent variables ($\mathbf{S}_{0,T}$). In order to solve this problem one defines the log-likelihood lower bound

$$\begin{aligned}\mathcal{F}^*(\tilde{p}, \theta) &= \langle \log p_\theta(\mathbf{X}_{0,T}) - D_{KL}(\tilde{p}(\mathbf{S}_{0,T}|\mathbf{X}_{0,T})|p_\theta(\mathbf{S}_{0,T}|\mathbf{X}_{0,T}))\rangle_{p^*(\mathbf{X}_{0,T})}, \\ &= \langle \log p_\theta(\mathbf{X}_{0,T}, \mathbf{S}_{0,T}) - \log \tilde{p}(\mathbf{S}_{0,T}|\mathbf{X}_{0,T})\rangle_{\tilde{p}(\mathbf{S}_{0,T}|\mathbf{X}_{0,T})p^*(\mathbf{X}_{0,T})}\end{aligned} \qquad [2]$$

where $\tilde{p}(\mathbf{S}_{0,T}|\mathbf{X}_{0,T})$ can be any (tractable) probability distribution, which in our case will be given through p_{dyn}. Finding maximum-likelihood parameters θ can then be done by iteratively maximizing $\mathcal{F}^*(\tilde{p}, \theta)$ with respect to $\tilde{p}$ (E-step) and θ (M-step). In the E-step p_θ is approximated by $\tilde{p}$ in order to estimate $\langle \log p_\theta(\mathbf{X}_{0,T}, \mathbf{S}_{0,T})\rangle_{\tilde{p}(\mathbf{S}_{0,T}|\mathbf{X}_{0,T})p^*(\mathbf{X}_{0,T})}$ and in the M-step this approximation is used to improve the model. This algorithm is guaranteed to converge to a local minimum, also if $\mathcal{F}^*$ is maximized only partially in every iteration, which makes it applicable to online learning.

Appealing to this theory in the following we show that: (i) Given a linear decoding model, a stochastic spiking neural network can be connected such that it can sample an efficient encoding online. This relates model- and network-parameters. (ii) The decoding model can be optimized online in respect to the sampled dynamics of the network. (iii) Combining (i) (the E-step) and (ii) (the M-step) yields update rules that can be applied by a stochastic spiking neural network to optimize its parameters in order to encode its inputs.

B.1. Online encoding by spiking neural network. Let us consider the following decoding model and prior on the spiking probability (Fig S1)

$$p_\theta(\mathbf{X}_{0,T}|\mathbf{S}_{0,T}) = \prod_t p_\theta(\mathbf{x}(t)|\mathbf{z}(t)) = \prod_t \mathcal{N}_{\mathbf{x}(t)}(D\mathbf{z}(t), \Sigma)$$

$$p_\theta(\mathbf{S}_{0,T}) = \prod_t p_\theta(\mathbf{s}(t)|\mathbf{z}(t)) = \prod_t \frac{1}{Z(\mathbf{b})} \exp\left(\mathbf{s}(t)^\top \mathbf{b}\right)$$

with $\Sigma = \sigma^2 I$ and parameters $\theta = \{D, \sigma, \mathbf{b}\}$. Notably this model asserts that at every time t, $\mathbf{x}(t)$ can be linearly decoded from spike traces $\mathbf{z}(t)$ with variance σ^2, where the spike traces are defined as before. Observe that the spike traces $\mathbf{z}(t)$ are deterministically defined given the preceding spikes $\mathbf{S}_{0,t-1}$. Also note that with the diagonal correlation matrix Σ, the decoder model assumes zero correlations between decoding errors. Input signals for which this assumption likely holds are for example signals with zero pairwise correlations between dimension, e.g. signals that have been whitened.

Since the model factorizes over time given the spike traces $\mathbf{z}(t)$, the log-likelihood lower bound (Eq 2) can be rewritten as

$$\begin{aligned}\mathcal{F}^*(p_{dyn}, \theta) &= \left\langle \sum_t \log p_\theta(\mathbf{x}(t), \mathbf{s}(t)|\mathbf{z}(t)) - \log p_{dyn}(\mathbf{s}(t)|\mathbf{x}(t), \mathbf{z}(t)) \right\rangle_{p_{dyn}(\mathbf{S}_{0,T}|\mathbf{X}_{0,T})p^*(\mathbf{X}_{0,T})} \\ &= \langle \log p_\theta(\mathbf{X}_{0,T})\rangle_{p^*(\mathbf{X}_{0,T})} - \\ &\quad \left\langle \sum_t \log p_{dyn}(\mathbf{s}(t)|\mathbf{x}(t), \mathbf{z}(t)) - \log p_\theta(\mathbf{s}(t)|\mathbf{X}_{t+1,T}, \mathbf{z}(t)) \right\rangle_{p_{dyn}(\mathbf{S}_{0,T}|\mathbf{X}_{0,T})p^*(\mathbf{X}_{0,T})}\end{aligned}$$

Here we substituted $\tilde{p}(\mathbf{S}_{0,T}|\mathbf{X}_{0,T}) = \prod_t p_{dyn}(\mathbf{s}(t)|\mathbf{x}(t), \mathbf{z}(t))$ and made use of the facts that spikes alter only the future decoding and that they are independent of the past given $\mathbf{z}(t)$, i.e. $p_\theta(\mathbf{s}(t)|\mathbf{X}_{0,T}, \mathbf{S}_{0,t-1}) = p_\theta(\mathbf{s}(t)|\mathbf{X}_{t+1,T}, \mathbf{z}(t))$.

We now perform the E-step. $\mathcal{F}^*$ is approximately maximized over p_{dyn} if $p_{\text{dyn}}(\mathbf{s}(t)|\mathbf{x}(t), \mathbf{z}(t)) \approx p_\theta(\mathbf{s}(t)|\mathbf{X}_{t+1,T}, \mathbf{z}(t))$ at every time t. However, this poses two problems:

(i) p_{dyn} depends only on $\mathbf{x}(t)$ while the spike probability in the model is based on future values $\mathbf{X}_{t+1,T}$, which are not available to the network.

(ii) In order to compute $p_\theta(\mathbf{s}(t)|\mathbf{X}_{t+1,T}, \mathbf{z}(t)) = \sum_{\mathbf{S}_{t+1,T}} p_\theta(\mathbf{S}_{t,T}|\mathbf{X}_{t+1,T}, \mathbf{z}(t))$ future spikes have to be marginalized out, which is intractable.

For the purpose of this paper we introduced simple approximations that solve these problems and work well in practice for our inputs. Specifically we assumed input- and network activity to be approximately constant over time. Hence all future inputs $\mathbf{x}(t') \in \mathbf{X}_{t+1,T}$ were assumed to be known to be $\mathbf{x}(t') = \mathbf{x}(t)$. Future network activity (independent of the current spike $\mathbf{s}(t)$) was assumed to be well approximated by a single trajectory, where neural outputs $\mathbf{z}(t)$ were constant. With this we can compute

$$
\begin{aligned}
&\sum_{\mathbf{S}_{t+1,T}} p_\theta(\mathbf{s}(t)|\mathbf{X}_{t+1,T}, \mathbf{z}(t), \mathbf{S}_{t+1,T}) p_\theta(\mathbf{S}_{t+1,T}|\mathbf{X}_{t+1,T}, \mathbf{z}(t)) \\
&\approx \prod_{t'=t}^{T} p_\theta\left(\mathbf{s}(t)|\mathbf{x}(t') = \mathbf{x}(t), \mathbf{z}(t') = \mathbf{z}(t) + \mathbf{s}(t)\exp\left(-\frac{t'-1-t}{\tau}\right)\right) \\
&= \frac{1}{Z(\theta,\mathbf{x})} \exp\left(\mathbf{s}(t)^\top \mathbf{b}\right) \prod_{t'=t+1}^{T} \exp\left(\frac{\mathbf{z}(t')^\top}{\sigma^2}[D^\top \mathbf{x}(t') - \frac{1}{2} D^\top D \mathbf{z}(t')]\right) \\
&= \frac{1}{Z(\theta,\mathbf{x})} \exp\left(\mathbf{s}(t)^\top \mathbf{b}\right) \prod_{t'=t+1}^{T} \exp\left(\frac{\left(\mathbf{z}(t) + \mathbf{s}(t)\exp\left(-\frac{t'-1-t}{\tau}\right)\right)^\top}{\sigma^2}\left[D^\top \mathbf{x}(t) - \right.\right. \\
&\qquad \left.\left. - \frac{1}{2} D^\top D \left(\mathbf{z}(t) + \mathbf{s}(t)\exp\left(-\frac{t'-1-t}{\tau}\right)\right)\right]\right) \\
&= \frac{1}{Z(\theta,\mathbf{x},\mathbf{z})} \exp\left(\mathbf{s}(t)^\top \left[\mathbf{b} + \frac{\tau}{\sigma^2} D^\top \mathbf{x}(t) - \frac{\tau}{\sigma^2} D^\top D \left(\mathbf{z}(t) + \frac{1}{4}\mathbf{s}(t)\right)\right]\right) \\
&= \frac{1}{Z(\theta,\mathbf{x},\mathbf{z})} \exp\left(\mathbf{s}(t)^\top \left[\mathbf{b} + \frac{\tau}{\sigma^2} D^\top \mathbf{x}(t) - \frac{\tau}{\sigma^2} D^\top D \mathbf{z}(t) - \frac{1}{4}\frac{\tau}{\sigma^2} \text{diag}(D^\top D)\right]\right)
\end{aligned}
$$

where we approximated $\sum_{t'=t+1}^{T} \exp(-\frac{t'-1-t}{\tau}) \approx \tau$ (that is τ and T large) and the last equality follows if timesteps are sufficiently small such that only one neuron spikes per timestep. Comparing with the network dynamics (Eq 1) from this we can conclude that a network that performs approximate online sampling from $p_\theta(\mathbf{S}_{0,T}|\mathbf{X}_{0,T})$ has parameters

$$
\begin{aligned}
F &= D^\top \\
W &= -D^\top D \\
T_j &= \frac{1}{4} W_{jj} - \frac{\sigma^2}{\tau} b_j \qquad [3] \\
\Delta u &= \frac{\sigma^2}{\tau}
\end{aligned}
$$

These results are similar to those yielded by a greedy spike encoding scheme (2). Please note that the sampling could be improved by using advanced sampling schemes, such as rejection sampling (6).

B.2. Online learning of an optimal decoder. As we have shown, the network dynamics implement an approximation of the E-step if the network parameters are chosen correctly. We will now use these samples produced by the network to incrementally improve the parameters of the decoding model in the M-step.

Recall that in the M-step we want to maximize under θ

$$
\left\langle \sum_t \log p_\theta(\mathbf{x}(t), \mathbf{s}(t)|\mathbf{z}(t)) \right\rangle_{p_{dyn}(\mathbf{S}_{0,T}|\mathbf{X}_{0,T}) p^*(\mathbf{X}_{0,T})}.
$$

Updates of the decoder model parameters should thus follow the gradient

$$
\Delta\theta = \tilde{\eta}_\theta \frac{\partial \mathcal{F}^*}{\partial \theta} = \tilde{\eta}_\theta \left\langle \sum_t \frac{\partial}{\partial \theta} \log p_\theta(\mathbf{x}(t), \mathbf{s}(t)|\mathbf{z}(t)) \right\rangle_{p_{dyn}(\mathbf{S}_{0,T}|\mathbf{X}_{0,T}) p^*(\mathbf{X}_{0,T})}
$$

In this paper we're only interested in the decoder weights D_{ij} from neuron j to input i, where the derivation yields

$$\Delta D_{ij} = \tilde{\eta}_D \left\langle \sum_t \sigma^{-2} z_j(t) \left(x_i(t) - \sum_k D_{ik} z_k(t) \right) \right\rangle_{p_{dyn}(\mathbf{S}_{0,T}|\mathbf{X}_{0,T})p^*(\mathbf{X}_{0,T})}$$

Here, $\tilde{\eta}_D$ is the update step size and σ^2 the variance of the decoder model. Note that in the following we will drop the dependence of the learning rate on σ^2, which has its motivation in covariant optimization (9). In covariant optimization, the gradient is multiplied by the inverse curvature of the loss function, because step size should be decreased when the curvature of the loss function is high. Since the curvature of the likelihood is proportional to the inverse variance, the variance drops out to yield a covariant gradient. This yields the update rule

$$\Delta D_{ij} = \tilde{\eta}_D \left\langle \sum_t z_j(t) \left(x_i(t) - \sum_k D_{ik} z_k(t) \right) \right\rangle_{p_{dyn}(\mathbf{S}_{0,T}|\mathbf{X}_{0,T})p^*(\mathbf{X}_{0,T})}$$

B.2.1. Online approximation. If many episodes $\mathbf{X}_{0,T}$ as sampled from $p^*(\mathbf{X}_{0,T})$ are presented in succession and spikes are sampled as outlined above, the average over samples from p_{dyn} can be replaced by an average over time

$$\left\langle \sum_t \cdot \right\rangle_{p_{dyn}(\mathbf{S}_{0,T}|\mathbf{X}_{0,T})p^*(\mathbf{X}_{0,T})} \approx \sum_t \langle \cdot \rangle_t \,.$$

If the update rules are performed every timestep this lets us rewrite them as

$$\delta D_{ij} = \eta_D z_j(t) \left(x_i(t) - \sum_k D_{ik} z_k(t) \right) \qquad [4]$$

This requires, however, that the learning rate η_D is sufficiently small such that changes in D_{ij} are negligible in a sufficiently long time window T'. In that case, summing the equation over time window T' yields

$$\begin{aligned} \sum_{t=0}^{T'} \delta D_{ij} &= \eta_D T' \left\langle z_j(t) \left(x_i(t) - \sum_k D_{ik} z_k(t) \right) \right\rangle_{t=0}^{T'} \\ &\overset{T' \to T}{\approx} \Delta D_{ij}, \end{aligned}$$

where the learning rates are related via $\tilde{\eta}_D = \eta_D T'$. A more refined statement can be made by rewriting the update equation as

$$\Delta D_{ij} = \tilde{\eta}_D \left(\langle z_j(t) x_i(t) \rangle_{p_{dyn}(\mathbf{S}_{0,T}|\mathbf{X}_{0,T})p^*(\mathbf{X}_{0,T})} - \sum_k D_{ik} \langle z_j(t) z_k(t) \rangle_{p_{dyn}(\mathbf{S}_{0,T}|\mathbf{X}_{0,T})p^*(\mathbf{X}_{0,T})} \right)$$

This makes explicit that the only information required to compute the gradient of the decoder weights are the correlations between neural outputs and inputs and in between neural outputs over the input sequences. Thus in practice, the learning rate η_D is ideally chosen as large as possible to allow fast learning, but also sufficiently small such that weight updates are performed with respect to a time window that provides a good estimate of correlations under the whole sampled spike trains.

B.3. Online learning of network parameters. So far we showed that the parameters of a network that samples from a decoder model are directly connected to the parameters of the model. We also showed how the decoder weights have to be updated such that they maximize the model likelihood over the generated samples. We will now combine these two results to find update rules for neural parameters directly, that can be used by neurons to learn an efficient encoding without supervision online. To this end we will first show how an approximation to the previously derived gradients can be implemented by regular stochastic LIF neurons. In a second step we will show how a better approximation can be realized by neurons with dendritic compartments. The central insight for all derivations will be that learning an E-I balance on membrane potentials corresponds to the learning of a decoder to the excitatory inputs times a transformation matrix that brings them into the space of the membrane potentials.

B.3.1. Somatic balance approximation.

Feedforward weights From the equality $F = D^\top$ (Eq 3) derived earlier and the update rule for D (Eq 4) we directly arive at

$$\delta F_{ji} = \eta_F z_j(t) \left(x_i(t) - \sum_k F_{ki} z_k(t) \right)$$

We follow previous approaches (2) and omit contributions to the decoding $\sum_k F_{ki} z_k(t)$ that are not available for the neuron, which is equivalent to assuming that neural spiking in the population is uncorrelated $\forall j \neq k : \langle z_j(t) z_k(t) \rangle_t \approx 0$. This yields the regularized Hebbian rule

$$\delta F_{ji} = \eta_F z_j(t) \left(x_i(t) - F_{ji} z_j(t) \right) \qquad [5]$$

Recurrent weights This rule will follow the optimal decoder gradient if spikes are indeed uncorrelated. However, if this is not the case the solution will be suboptimal and furthermore the previously derived recurrent weights $W = -D^\top D$ together with the suboptimal weights F does not enable a reasonable encoding anymore. Both problems can be addressed by observing that for the optimal membrane potential we derived

$$\mathbf{u}^{opt}(t) = D^\top \mathbf{x}(t) - D^\top D\mathbf{z}(t) = D^\top(\mathbf{x}(t) - D\mathbf{z}(t)),$$

i.e. the potentials are proportional to the decoding error. This can be approximately guaranteed even if the feedforward weights are suboptimal (but not zero) by setting $W = -FD$, since then

$$\mathbf{u}(t) = F\mathbf{x}(t) - FD\mathbf{z}(t) = F(\mathbf{x}(t) - D\mathbf{z}(t)) \overset{\approx}{\propto} \mathbf{u}^{opt}(t).$$

To make sure that neurons adapt their encoding for an optimal decoder, recurrent weights will adapt along the gradient of decoder weights. For fixed encoder weights F this yields

$$\begin{aligned}\delta W_{jk} &= -\sum_i F_{ji}\delta D_{ik} \\ &= -\eta_W z_k(t)\left(\sum_i F_{ji}x_i(t) - \sum_{i,l} F_{ji}D_{il}z_l(t)\right) \\ &= -\eta_W z_k(t)\left(\sum_i F_{ji}x_i(t) + \sum_l W_{jl}z_l(t)\right) \\ &= -\eta_W z_k(t)u_j(t)\end{aligned} \qquad [6]$$

This shows that through an E-I balance, this rule for W self-consistently finds the correct decoder 'inside' of the recurrent weights, and hence allows the projection of the right decoding error $x - Dz$. Thereby recurrent connections ensure a reasonable encoding even if feedforward weights are not learned optimally. Since in the equation above F_{ji} is assumed constant, we chose the learning rate η_W 2-4 times larger than η_F. In simulations we found that recurrent weights that evolve under Eq 6 indeed converged to $W = -FD$, where D is the optimal decoder weights obtained under the non-local update rule Eq 4 .

B.3.2. Learning encoder weights with dendritic balance . In the following we devise examples for local plasticity rules for feedforward inputs that follow the correct gradient of the likelihood lower bound. Locality requires that the decoding of other neurons is made available at the synapse, which can then be used to find the correct gradient. We argue that this can be mediated by dendritic recurrent connections W^i that target dendrites where the feedforward input i has formed a synapse. Due to strong attenuation between dendritic compartments, the membrane potential u_j^i in the vicinity of synapse i on that dendrite only integrates inputs that are present locally, i.e.

$$u_j^i(t) = \underbrace{F_{ji}x_i(t)}_{\text{feedforward input}} + \underbrace{\textstyle\sum_k W_{jk}^i z_k(t)}_{\text{recurrent input}}. \qquad [7]$$

We then assume a regime where currents from the dendrites are summed linearly, such that the total membrane potential at the soma is given by $u_j(t) = \sum_i u_j^i(t)$. Similar to recurrent somatic connections, we will show that recurrent dendritic connections can locally learn an optimal decoding of neural PSPs $\mathbf{z}$ by enforcing *dendritic balance* of feedforward and recurrent inputs. The central feature of this approach is that feedforward and recurrent connections both use the dendritic potential for learning, which requires their cooperation. We here show three possible mechanisms that realize this and yield very similar behaviour to the analytical solution (Fig S2, S3).

Slow feedforward adaptation One possibility to ensure the cooperation of feedforward and recurrent weights is to separate the timescales on which they are adapting. To that end we make the optimal ansatz for recurrent weights similar to before $W_{jk}^i = -F_{ji}D_{ik}$. Then, changing recurrent weights in the direction of the decoder gradient of Eq 4 yields

$$\begin{aligned}\delta W_{jk}^i &= -F_{ji}\delta D_{ik} \\ &= -\eta_W z_k(t)(F_{ji}x_i(t) - \textstyle\sum_l F_{ji}D_{il}z_l(t)) \\ &= -\eta_W z_k(t)(F_{ji}x_i(t) + \textstyle\sum_l W_{jl}^i z_l(t)) \\ &= -\eta_W z_k(t)u_j^i(t).\end{aligned}$$

where we again assumed that changes in feedforward weights are slow and can be neglected, and $\eta_W = \eta_D$. We conclude that enforcing dendritic balance by recurrent plasticity is equivalent to locally optimizing a decoder $D_{ik} = -W_{jk}^i/F_{ji}$.

The correct gradient of the decoder weights can also be calculated locally, but it can't be applied to the feedforward weights directly since this would contradict the previously made assumption of slow changes in feedforward weights. However, it is

possible to locally integrate the correct gradient and use this to adapt feedforward weights slowly, with a delay. To this end we introduce the local integration variable $I_{ji} = F_{ji}D_{ij}$, which adapts according to the decoder gradient times F_{ji}

$$\begin{aligned}\delta I_{ji} =& \eta_I F_{ji} z_j(t) \left(x_i(t) - \textstyle\sum_k D_{ik} z_k(t) \right) \\ =& \eta_I z_j(t) u_j^i(t),\end{aligned} \tag{8}$$

with $\eta_I = \eta_D$. F_{ji} then can slowly follow D_{ij} via

$$\delta F_{ji} = \eta_F (I_{ji}/F_{ji} - F_{ji}),$$

with $\eta_F \ll \eta_D$. Note that for $F_{ji} = 0$ the gradient for F_{ji} is not defined. In this case the learning process could be kickstarted via simple Hebbian learning on F_{ji}. Note also that the equation $W^i_{jk} = -F_{ji}D_{ik}$ has to hold at the start of learning, which can be guaranteed by simply choosing $W^i_{jk} = F_{ji} = 0$. To summarize, slow feedforward adaptation leads to neural parameters $W^i_{jk} = -F_{ji}D_{ik}$ and $F_{ji} = D_{ij}$. This shows that feedforward synapses slowly can evolve to minimize the decoder error along its gradient using only local information.

Simultaneous adaptation of feedforward and recurrent weights In principle it would also be possible to adapt feedforward and recurrent weights simultaneously without a separation of timescales. However, calculating the gradient for the derived recurrent weights is locally not feasible, since we find

$$\begin{aligned}\delta W^i_{jk} =& - D_{ji}\delta D_{ik} - \delta D_{ji} D_{ik} \\ =& - \eta_D (z_k(t) u^i_j(t) + z_j(t) u^i_k(t)).\end{aligned}$$

Empirically we found that the contralateral contributions $z_j(t)u^i_k(t)$ to the gradient can be approximated by the accessible contributions $z_k(t)u^i_j(t)$. We thus approximate $\langle z_j(t)u^i_k(t)\rangle_t \approx \langle z_k(t)u^i_j(t)\rangle_t$. While this equation does not hold for all i, j, k, we still find that the resulting learned contributions to the dendritic potentials have the correct magnitude, hence enabling feedforward learning. The gradient for the recurrent weights now are

$$\delta W^i_{jk} = - \eta_W z_k(t) u^i_j(t),$$

where $\eta_W = 2\eta_D$.

Assuming the correct recurrent weights $W^i_{jk} = -D_{ji}D_{ki}$ we can find the decoded population encoding locally at the dendrite. From the self-consistency $F_{ji} = D_{ij}$ and Eq 7 we have the relation

$$\sum_k D_{jk} z_k(t) = \frac{F_{ji} x_i(t) - u^i_j(t)}{F_{ji}}.$$

With this we can implement the learning of feedforward weights in way that highlights its similarity to previous approaches (Eq 5)

$$\delta F_{ji} = \eta_F z_j(t) \left(x_i(t) - \frac{F_{ji} x_i(t) - u^i_j(t)}{F_{ji}} \right),$$

i.e. the rule is a regularized Hebbian plasticity rule. Again, for very small F_{ji} the regularization becomes unstable, but can be left away (since it should go to zero) leaving a purely Hebbian rule. For the derivation we used $\eta_F = \eta_D$, which implies that we should choose $\eta_W \approx 2\eta_F$.

In simulations we verified that the approximations we made for this learning scheme are adequate and yield feed forward weights for which $F_{ji} = D_{ij}$ holds with high accuracy. Note that the network found by the presented learning scheme only corresponds to the decoding model if $\eta_W \approx 2\eta_F$. However, if the recurrent learning is faster this only results in a rescaling of feedforward weights by a factor of $2\eta_F/\eta_W$, since their adaptation is too slow by this factor. This means that in this case the "correct" dynamics of the network can be recovered via a rescaling of all weights, or equivalently, with firing rate adaptation a change in the stochasticity of spiking Δu by a factor of $2\eta_F/\eta_W$.

Learning of feedforward and recurrent weights via the weight decay trick For both learning schemes we have presented so far, the relation of feedforward and recurrent weights and their learning rates $\eta_{(\cdot)}$ are critical for learning, as changes in recurrent weights directly impact how feedforward weights are learned and vice versa. This can become problematic, if the recurrent weights are not initialized as $W^i_{jk} = -F_{ji}F_{ki}$ or if for some reason the match of feedforward and recurrent weights is disturbed during learning. This problematic co-dependence of the learning rules can be avoided via a simple trick, which we will call the weight decay trick. To this end we introduce a small weight decay with rate λ_j on the decoder weights

$$\delta D_{ij} = \eta_D z_j(t) \left(x_i(t) - \sum_k D_{ik} z_k(t) \right) - \lambda_j D_{ij}. \tag{9}$$

By doing so, we can readily derive an implicit equation for the fixed point of this update rule, which is

$$D^*_{ij} = \left\langle \lambda_j^{-1} z_j(t) \left(x_i(t) - \sum_k D^*_{ik} z_k(t) \right) \right\rangle_t = \left\langle \lambda_j^{-1} F_{ji}^{-1} z_j(t) u^i_j(t) \right\rangle_t.$$

This equation holds if recurrent weights were learned to approximately equal $W^i_{jk} = -F_{ji} D^*_{ik}$. This can be achieved by updating recurrent weights until convergence with

$$\begin{aligned} \delta W^i_{jk} &= -F_{ji} \delta D_{ik} \\ &= -\eta_W \left(z_k(t) u^i_j(t) - \lambda_k W^i_{jk} \right) . \end{aligned}$$

Now the optimal feedforward weights can be learned by slowly tracking the fixed point D^*_{ij}, which can be computed locally

$$\begin{aligned} \delta F_{ji} &= \eta_F \lambda_j \left(D^*_{ij} - F_{ji} \right) \\ &\approx \eta_F \left(F^{-1}_{ji} z_j(t) u^i_j(t) - \lambda_j F_{ji} \right) . \end{aligned}$$

Here the pre-factor λ_j normalizes the learning speed. Interestingly, this learning rule is simply the gradient for feedforward weights, as calculated before, with additional weight-decay similar to the recurrent learning rule. The difference of this learning scheme to the previous two learning schemes is that inhibition will not perfectly balance excitation, but the balance will be offset by a small amount. Feedforward learning then relies on this small mismatch between excitation and inhibition to find a good encoding and thereby avoids the problematic co-dependence of feedforward and recurrent learning.

How does this learning scheme, which evidently relies on a different decoder update, relate to the previously derived network dynamics corresponding to the optimal decoder? A valid concern would be that an offset in the E-I balance could lead to elevated or reduced spiking rates. The answer is that there exists a close relation between the weight decay λ_j and the spiking prior b_j, which helps to ensure optimal spiking. More technically, the weight decay of the decoder can be seen as a constraint on the L2-norm of decoder weights, to compensate for a fixed, sub-optimal threshold. To understand this, we start with the equation for the optimal threshold T_j (Eq 3). If the threshold T_j is fixed to an arbitrary value, this equation directly implies a length constraint on the decoder

$$\begin{aligned} T_j &= -\frac{1}{4} \sum_i D^2_{ij} - \frac{\sigma^2}{\tau} b_j \\ \Leftrightarrow \sum_i D^2_{ij} &= -4T_j - \frac{4\sigma^2}{\tau} b_j \stackrel{\text{def}}{=} a_j . \end{aligned}$$

This means that neurons can not only fire optimally for a given prior b_j by changing their thresholds in accordance with the strength of incoming connections, but also by changing the overall connection strength while keeping the threshold fixed. This constraint can be included into the optimization by augmenting the decoder loss (containing all relevant contributions of the likelihood, Eq 2) via Lagrangian optimization

$$\mathcal{L}(D) = \frac{1}{2} \left\langle ||\mathbf{x}(t) - D\mathbf{z}(t)||^2 \right\rangle_t + \sum_j \lambda_j \frac{1}{2} \left(||D_j||^2 - a_j \right) .$$

From this loss the decoder update with weight decay (Eq 9) directly arises via gradient descent. Here, the Lagrangian multipliers λ_j correspond to specific firing priors of neurons b_j for some fixed threshold T_j. It is therefore possible to obtain correct network dynamics by either adapting λ_j according to $\delta\lambda_j \propto -\frac{\partial \mathcal{L}}{\partial \lambda_j}$, or by simply treating λ_j as a parameter of the model instead of b_j. It is therefore also evident that changes in network dynamics in comparison to the analytical network (Eq 3) are minimal as long as the λ_j are small. Especially with additional rapid firing rate adaptation, which we are using in our simulations, the difference to the analytical solution is negligible for small λ_j, as here the impact of λ_j on the firing rate is 'overwritten' by the rapidly adapting threshold.

B.3.3. Rapid firing rate adaptation. In the Bayesian framework Habenschuss and colleagues have shown that a rapid rate adaptation can be interpreted as a constraint on the variational approximation in the E-step (10). For the resulting constrained optimization formally a Lagrange multiplier is introduced which 'overwrites' the analytic threshold $T_j = \frac{1}{4} W_{jj} - \sigma^2 \tau^{-1} b_j$. We will not make a notational difference between the two thresholds here. The fixed firing rate is enforced by adapting the threshold T_j such that neurons are firing with a target firing rate ρ.

$$\delta T_j = \eta_T (s_j - \rho\, \delta)$$

Here, $\rho\,\delta$ is the mean number of spikes in a time window of size δ if a neuron would spike with rate ρ and s_j is a spike indicator which is 1 if z_j spiked in the last time δ, otherwise $s_j = 0$. Since this is a constraint that is applied in the E-step, the learning rate η_T should be large.

B.3.4. Pruning recurrent weights. In the proposed learning schemes the number of recurrent connections grows very fast with network and input size (# of inh. conn. $= N_x \times N_z^2$). We here propose a principle by which recurrent connections that provide little contribution to neural learning can be pruned away (Please note that the following principle only considers *learning*; for correct *dynamics* it might be necessary to keep additional somatic weights that ensure efficient spiking). To identify these weights we again look at the learning rule of the proposed slow feedforward weight adaptation scheme (Eq 8)

$$\begin{aligned}\delta I_{ji} =& \eta_I z_j(t) u_j^i(t) \\ =& \eta_I z_j(t) \left(F_{ji} x_i(t) + \sum\nolimits_k W_{jk}^i z_k(t) \right) .\end{aligned}$$

Here, recurrent connections that provide no systematic contribution to the gradient can be left away. In particular, those are connections W_{jk}^i for which $\langle z_j(t) W_{jk}^i z_k(t) \rangle_t \approx 0$. In other words, only large weights matter that connect neurons with correlated activities. Hence, the number of required weights for learning depends primarily on the sizes of neural receptive fields (as $W_{jk}^i \approx -F_{ji} F_{ki}$) and the number of correlated coding neurons and not the size of the network and input.

Based on this observation, one possibility to prune weights is for example to remove a certain fraction of the weights and leaving only the weights with the largest $|\langle z_j(t) W_{jk}^i z_k(t) \rangle_t|$. In biological neurons potential connections W_{jk}^i could continuously be probed and only be stably formed if their contribution is sizeable. For the bar task, we demonstrated that this allows us to prune a very large fraction of recurrent weights without compromising performance (Fig S9).

It is important to note that in no case feedforward weights should remain un-regularized, that is, the learning rule is purely Hebbian, as this would lead to unbounded growth of weights. The best solution to this problem is to always keep self-contributions to the gradient $W_{jj}^i z_j \approx -F_{ij} F_{ij} z_j$. This results in the same regularization as it is used in the somatic balance model and can arguably be always computed locally.

C. Relation to previous studies of representation learning

C.1. Comparison to other Hebbian-like learning rules. The Hebbian-like learning rule used in the somatic balance model is part of a group of Hebbian-like learning rules that have been used in the past to model representation learning in recurrent populations of neurons. We here present a (non-exhaustive) overview over such rules that learn feedforward weights F_{ji} (Table S1). All these rules can be seen as successors of the well known Oja's rule (11), which can be written in the form

$$\Delta F_{ji} \propto z_j(x_i - F_{ji} z_j),$$

where x_i is some input and $z_j = \sum_i F_{ji} x_i$ is the activity of a (linear) neuron. Specifically, all rules we will present can be written in the more general form

$$\Delta F_{ji} \propto \text{post} \times (\text{pre} - f(F_{ji}) \times \text{post}),$$

where "post" and "pre" denote aspects of post- and presynaptic activity, respectively, and $f(\cdot)$ is some function of the weight. We will write $s_j \in \{0, 1\}$ to denote a binary spike indicator and z_j to denote some form of analog postsynaptic activity.

Note that by itself Oja's rule always extracts the largest principal component of the input data x_i. This means that in order to learn non-redundant representations in a network, some form of recurrent inhibitory coupling is required. Importantly, as we have argued, in order to be generally applicable it requires inhibition that is nearly instantaneous and therefore biologically implausible. Consequently, most models we present here make use of some form of instantaneous (or implausible) inhibition. Some of the models get around this constraint by other means, e.g. by forcing zero correlations in an extremely slow-firing regime (12), or have only been tested for very simple scenarios (13).

Table S1. List of related papers modeling representation learning with Hebbian-like plasticity.

Reference	Rule	Comment
Foeldiak (1990) (14)	$\Delta F_{ji} \propto s_j(x_i - F_{ji} s_j)$	This paper uses binary neurons, where outputs s_j are determined by an optimization scheme.
Kung et al (1990) (15)	$\Delta F_{ji} \propto z_j(x_i - F_{ji} z_j)$	This paper uses linear neurons, where outputs z_j are determined in a strictly sequential manner.
Zylberg et al (2011) (12)	$\Delta F_{ji} \propto z_j(x_i - F_{ji} z_j)$	z_j is a spike-counter over a certain time window. This paper uses LIF neurons with recurrent inhibition in a slow-firing regime.
Kappel et al (2014) (6) (similarly (5))	$\Delta F_{ji} \propto s_j(x_i - e^{F_{ji}} s_j)$	To achieve "canonical" form we multiplied the rule with $e^{F_{ji}}$, changing the learning speed, but not the fixed point. This paper uses stochastic spiking neurons in a winner-take-all circuit.
Bill et al (2015) (4)	$\Delta F_{ji} \propto s_j(x_i - \text{sig}(F_{ji} + F_{0i}) s_j)$	$\text{sig}(\cdot)$ is the logistic function and F_{0i} is a baseline constant. This paper uses stochastic spiking neurons in a winner-take-all circuit.
Bahroun et al (2017) (16)	$\Delta F_{ji} \propto [\sum_{t'}^{t} z_j(t')^2]^{-1} z_j(x_i - F_{ji} z_j)$	Learning speed is regulated with a pre-factor. This paper uses analog neurons, where outputs z_j are the results of an optimization scheme.
Pehlevan et al (2017) (17)	$\Delta F_{ji} \propto [\sum_{t'}^{t} z_j(t')^2]^{-1} z_j(x_i - F_{ji} z_j)$	This paper proposes a network with very similar behavior to (16), but performs non-negative source separation.
Jonke et al (2017) (13)	$\Delta F_{ji} \propto s_j(x_i - \text{sig}(\gamma F_{ji}) s_j)$	$\text{sig}(\cdot)$ is the logistic function and γ is a scaling parameter. This paper uses stochastic spiking neurons in a k-winner-take-all circuit.
Tavanei et al (2018) (18)	$\Delta F_{ji} \propto s_j(x_i - (1 - \lambda) F_{ji} s_j)$	λ is a sparsity factor. This paper uses spiking neurons in a winner-take-all circuit.
Brendel et al (2020) (2)	$\Delta F_{ji} \propto s_j(x_i - \alpha F_{ji} s_j)$	α is some regularization factor. This paper uses LIF neurons with recurrent inhibition and noisy potentials, resulting in a model that is practically identical to ours. Additionally, only one neuron is allowed to spike per time-bin.
This paper	$\Delta F_{ji} \propto z_j(x_i - F_{ji} z_j)$	This paper uses stochastic LIF neurons with recurrent inhibition and spike traces z_j.

C.2. Comparison to Brendel et al (2020). Our neural model and the model used by Brendel et al (2) are practically identical. Both models employ stochastic leaky integrate-and-fire neurons, which can be seen as instances of the spike response model with escape noise (19). Brendel et al employ a formulation with partial differential equations, while we use a formulation where the shape of PSP's is solved. Brendel et al add stochasticity to neural firing by adding Gaussian noise to the membrane potential, while we directly write down a probability function for spiking. Overall, this results in a stochastic neuron that is approximately equal to our probabilistic formulation (see e.g. (19), chapter 9.4).

The goal of coding is the same in both models. Hence, the feedforward learning rule of the somatic balance model is also, for all practical purposes in this paper, the same as it has been used by Brendel et al, which reads

$$\delta F_{ji} \propto s_j(t)\left(x_i(t) - \alpha F_{ji} s_j(t)\right), \tag{10}$$

where α is some regularization factor. Notably, this rule only updates weights on spike-times, whereas our Hebbian-like rule (Eq 5) also incorporates non-spike-times into the update (the non-spike-times are an essential contribution in the dendritic balance learning scheme). For constant $x_i(t)$, which we use in our simulations, our rule can be integrated over time for a single spike $z_j(t) = s_j(t_s)\exp\left(-\frac{t-t_s}{\tau}\right)$ at time t_s:

$$\begin{aligned} \eta_F \int_{t=t_s}^{\infty} s_j(t_s)\exp\left(-\frac{t-t_s}{\tau}\right)\left(x_i(t) - F_{ji}s_j(t_s)\exp\left(-\frac{t-t_s}{\tau}\right)\right) dt \\ = \eta_F \tau s_j(t_s)\left(x_i(t) - 0.5F_{ji}s_j(t_s)\right). \end{aligned} \tag{11}$$

Hence, when spikes are rare events our rule is the same as the rule by Brendel et al, with $\alpha = 0.5$ and the learning rate η_F chosen appropriately. For fast spiking neurons the regularization is slightly different, since past spikes are taken into account when regularizing the weight. However, the overall learning outcome will be very similar since this only slightly changes the magnitude of the weight-vector. To verify this we adapted their implementation of the network (20), and found that the major effects we report in Fig 5F-H (SB) are preserved.

D. Datasets

MNIST The standard MNIST database of handwritten digits was used (21). Images were scaled down from 28×28 to 16×16 pixels. No further preprocessing was applied.

Correlated bars See description in Fig 4A. Pixels where bars are displayed (also in the case of overlap) were set to the value 1.0, pixels without bars were set to 0.0.

Natural scenes Images for natural scenes were taken from (22). A simple preprocessing was applied to ensure that they can be modeled by spiking neurons. Importantly we required that input is always positive. Every image χ in the database was whitened. This can be seen as an approximation of retinal on/off-cell preprocessing, where one on-cell and one off-cell with overlapping fields are lumped together in a single value χ_i, which can be positive or negative. We separated every value χ_i into two values $x'_{2i} = \chi_i$ and $x'_{2i+1} = -\chi_i$. We then applied a continuous nonlinear activation function to ensure that activations are positive and bimodally distributed (i.e. mostly close to either 0.0 or 1.0): $x_i = \mathrm{sig}(3.2(x'_i - 0.8))$, where $\mathrm{sig}(x) = 1/(1 + \exp(-x))$. For the display of learned weights we merge corresponding values again and display $x_{2i} - x_{2i+1}$.

Speech The speech data-set is the same as used in (2) and was taken from (20). The speech signal was presented in a spectral decomposition with 25 frequency channels, sampled at 200Hz with linear interpolation between data-points. The signal was spatially whitened using Cholesky whitening. After whitening we applied the same splitting and rectification procedure as for the natural scenes input signals.

In contrast to our results in Fig 5F-H, the original task in (2) uses the unwhitened signal directly as input. For this unwhitened input, (2) show that the somatic balance model requires a learning rule that - additionally to the Hebbian-like learning - performs spatial whitening to remove pairwise correlations in the signal. To check that our pre-processing does not significantly alter the results, we also performed simulations without whitening and using this alternative learning rule as used by (2) (Fig S11), and observed similar behavior as in Fig 5F-H.

E. Parameters

For all tasks parameters were tuned to ensure that networks operate well. *DB* denotes networks where the analytic solution given the decoder was used for network dynamics. *DB slow* are networks with slow feedforward adaptation, *DB simultaneous* are networks with parallel adaptation of feedforward and recurrent weights. *SB* are networks learning with somatic balance. When the parameter $\eta_{\Delta u}$ is present the stochasticity of spiking was annealed starting from 1.0 with rate $\eta_{\Delta u}$. Learning rates η_θ are given in units of ms^{-1}. Networks in all simulations were initialized with zero initial weights, except for Figs 3 and 5C-E, where feedforward weights were initialized as $F_{ji} = \exp(\max(0, 0.3 \cdot r_{ji} - 0.2)) - 1$ with $r_{ji} \sim \mathcal{N}(0, 1)$.

MNIST (Fig 3, 5)

Parameter	DB decay	SB
δ	0.1ms / 0.3ms	0.1ms / 0.3ms
τ	10ms	10ms
Δu	0.1	0.1
ρ	15s^{-1}	15s^{-1}
λ	0.03	-
η_T	$7.0 \cdot 10^{-3}$	$7.0 \cdot 10^{-3}$
η_F	$1.5 \cdot 10^{-5}$	$4.0 \cdot 10^{-6}$
η_W	$3.0 \cdot 10^{-5}$	$3.0 \cdot 10^{-5}$
η_D	$1.0 \cdot 10^{-6}$	$1.0 \cdot 10^{-6}$

MNIST (Fig S2)

Parameter	DB	DB simultaneous	DB slow	DB decay	SB
δ	0.1ms	0.1ms	0.1ms	0.1ms	0.1ms
τ	10ms	10ms	10ms	10ms	10ms
Δu	0.1	0.1	0.1	0.1	0.1
ρ	20s^{-1}	20s^{-1}	20s^{-1}	20s^{-1}	20s^{-1}
λ	-	-	-	0.005	-
η_T	$5.0 \cdot 10^{-3}$	$3.0 \cdot 10^{-3}$	$5.0 \cdot 10^{-4}$	$5.0 \cdot 10^{-4}$	$5.0 \cdot 10^{-3}$
η_F	$5.0 \cdot 10^{-6}$	$3.0 \cdot 10^{-6}$	$4.0 \cdot 10^{-7}$	$2.0 \cdot 10^{-6}$	$5.0 \cdot 10^{-6}$
η_I	-	-	$4.0 \cdot 10^{-5}$	-	
η_W	-	$6.0 \cdot 10^{-6}$	$4.0 \cdot 10^{-5}$	$6.0 \cdot 10^{-5}$	$1.0 \cdot 10^{-5}$
η_D	$5.0 \cdot 10^{-6}$	$3.0 \cdot 10^{-6}$	$5.0 \cdot 10^{-6}$	$5.0 \cdot 10^{-6}$	$5.0 \cdot 10^{-6}$

Correlated bars (Fig 4, S3, S9)

Parameter	DB	DB simultaneous	DB slow	DB decay	SB
δ	1.0ms	1.0ms	1.0ms	1.0ms	1.0ms
τ	10ms	10ms	10ms	10ms	10ms
Δu^*	0.1	0.1	0.1	0.1	0.1
$\eta_{\Delta u}$	$7.0 \cdot 10^{-8}$	$7.0 \cdot 10^{-8}$	$7.0 \cdot 10^{-8}$	$7.0 \cdot 10^{-8}$	$7.0 \cdot 10^{-8}$
ρ	15s^{-1}	15s^{-1}	15s^{-1}	15s^{-1}	15s^{-1}
λ	-	-	-	0.005	-
η_T	$1.0 \cdot 10^{-2}$	$1.0 \cdot 10^{-2}$	$5.0 \cdot 10^{-2}$	$5.0 \cdot 10^{-2}$	$1.0 \cdot 10^{-2}$
η_F	$5.0 \cdot 10^{-5}$	$5.0 \cdot 10^{-5}$	$1.0 \cdot 10^{-7}$	$2.0 \cdot 10^{-5}$	$5.0 \cdot 10^{-5}$
η_I	-	-	$5.0 \cdot 10^{-5}$	-	-
η_W	-	$1.0 \cdot 10^{-4}$	$5.0 \cdot 10^{-5}$	$1.0 \cdot 10^{-4}$	$1.0 \cdot 10^{-4}$
η_D	$5.0 \cdot 10^{-5}$	$5.0 \cdot 10^{-5}$	$5.0 \cdot 10^{-5}$	$5.0 \cdot 10^{-5}$	$5.0 \cdot 10^{-5}$

Natural scenes (Fig 4, 5, S4, S5, S6, S7, S8, S10)

Parameter	DB	SB
δ	0.2ms	0.2ms
τ	10ms	10ms
Δu^*	0.13	0.13
$\eta_{\Delta u}$	$7.0 \cdot 10^{-8}$	$7.0 \cdot 10^{-8}$
$\rho \cdot$ # neurons	1000s^{-1}	1000s^{-1}
η_T until $t = 6000$s	$6.0 \cdot 10^{-3}$	$6.0 \cdot 10^{-3}$
η_T until $t = \infty$	$4.0 \cdot 10^{-3}$	$4.0 \cdot 10^{-3}$
η_F until $t = 6000$s	$4.0 \cdot 10^{-5}$	$4.0 \cdot 10^{-5}$
η_F until $t = \infty$	$4.0 \cdot 10^{-5}$	$3.0 \cdot 10^{-5}$
η_W until $t = 6000$s	-	$10.0 \cdot 10^{-5}$
η_W until $t = \infty$	-	$7.0 \cdot 10^{-5}$
η_D until $t = 6000$s	$4.0 \cdot 10^{-5}$	$4.0 \cdot 10^{-5}$
η_D until $t = \infty$	$3.0 \cdot 10^{-5}$	$3.0 \cdot 10^{-5}$

Speech (Fig 5, S11)

Parameter	DB	SB
δ	0.05ms	0.05ms
τ	10ms	10ms
Δu	0.05	0.05
ρ	5s^{-1}	5s^{-1}
η_T	$1.4 \cdot 10^{-2}$	$1.4 \cdot 10^{-2}$
η_F	$2.1 \cdot 10^{-4}$	$2.1 \cdot 10^{-4}$
η_W	-	$5.6 \cdot 10^{-4}$
η_D	$2.1 \cdot 10^{-4}$	$2.1 \cdot 10^{-4}$

References

1. W Gerstner, WM Kistler, *Spiking neuron models: Single neurons, populations, plasticity.* (Cambridge university press), (2002).
2. W Brendel, R Bourdoukan, P Vertechi, CK Machens, S Denéve, Learning to represent signals spike by spike. *PLoS computational biology* **16**, e1007692 (2020).
3. B Nessler, M Pfeiffer, L Buesing, W Maass, Bayesian Computation Emerges in Generic Cortical Microcircuits through Spike-Timing-Dependent Plasticity. *PLoS Comput. Biol.* **9**, e1003037 (2013).
4. J Bill, et al., Distributed Bayesian Computation and Self-Organized Learning in Sheets of Spiking Neurons with Local Lateral Inhibition. *PLOS ONE* **10**, e0134356 (2015).
5. B Nessler, M Pfeiffer, W Maass, Stdp enables spiking neurons to detect hidden causes of their inputs in *Advances in neural information processing systems.* pp. 1357–1365 (2009).
6. D Kappel, B Nessler, W Maass, STDP Installs in Winner-Take-All Circuits an Online Approximation to Hidden Markov Model Learning. *PLoS Comput. Biol.* **10**, e1003511 (2014).
7. S Deneve, Bayesian Spiking Neurons II: Learning. *Neural Comput.* **20**, 118–145 (2007).
8. RM Neal, GE Hinton, A view of the em algorithm that justifies incremental, sparse, and other variants in *Learning in graphical models.* (Springer), pp. 355–368 (1998).
9. DJ MacKay, *Information theory, inference and learning algorithms.* (Cambridge university press), (2003).
10. S Habenschuss, J Bill, B Nessler, Homeostatic plasticity in bayesian spiking networks as expectation maximization with posterior constraints in *Advances in neural information processing systems.* pp. 773–781 (2012).
11. E Oja, Simplified neuron model as a principal component analyzer. *J. mathematical biology* **15**, 267–273 (1982).
12. J Zylberberg, JT Murphy, MR DeWeese, A sparse coding model with synaptically local plasticity and spiking neurons can account for the diverse shapes of v1 simple cell receptive fields. *PLoS Comput. Biol* **7**, e1002250 (2011).
13. Z Jonke, R Legenstein, S Habenschuss, W Maass, Feedback inhibition shapes emergent computational properties of cortical microcircuit motifs. *J. Neurosci.* **37**, 8511–8523 (2017).
14. P Földiak, Forming sparse representations by local anti-hebbian learning. *Biol. cybernetics* **64**, 165–170 (1990).
15. SY Kung, KI Diamantaras, A neural network learning algorithm for adaptive principal component extraction (apex) in *International Conference on Acoustics, Speech, and Signal Processing.* (IEEE), pp. 861–864 (1990).
16. Y Bahroun, A Soltoggio, Online representation learning with single and multi-layer hebbian networks for image classification in *International Conference on Artificial Neural Networks.* (Springer), pp. 354–363 (2017).
17. C Pehlevan, S Mohan, DB Chklovskii, Blind nonnegative source separation using biological neural networks. *Neural computation* **29**, 2925–2954 (2017).
18. A Tavanaei, T Masquelier, A Maida, Representation learning using event-based stdp. *Neural Networks* **105**, 294–303 (2018).
19. W Gerstner, WM Kistler, R Naud, L Paninski, *Neuronal dynamics: From single neurons to networks and models of cognition.* (Cambridge University Press), (2014).
20. C Machens, Github - machenslab/spikes (https://github.com/machenslab/spikes) (2020) Online; accessed 2021-02-20.
21. Y LeCun, C Cortes, C Burges, Mnist handwritten digit database (http://yann.lecun.com/exdb/mnist) (2010) Online; accessed 2020-05-20.
22. B Olshausen, Sparse coding simulation software (http://www.rctn.org/bruno/sparsenet/) (1996) Online; accessed 2020-05-20.

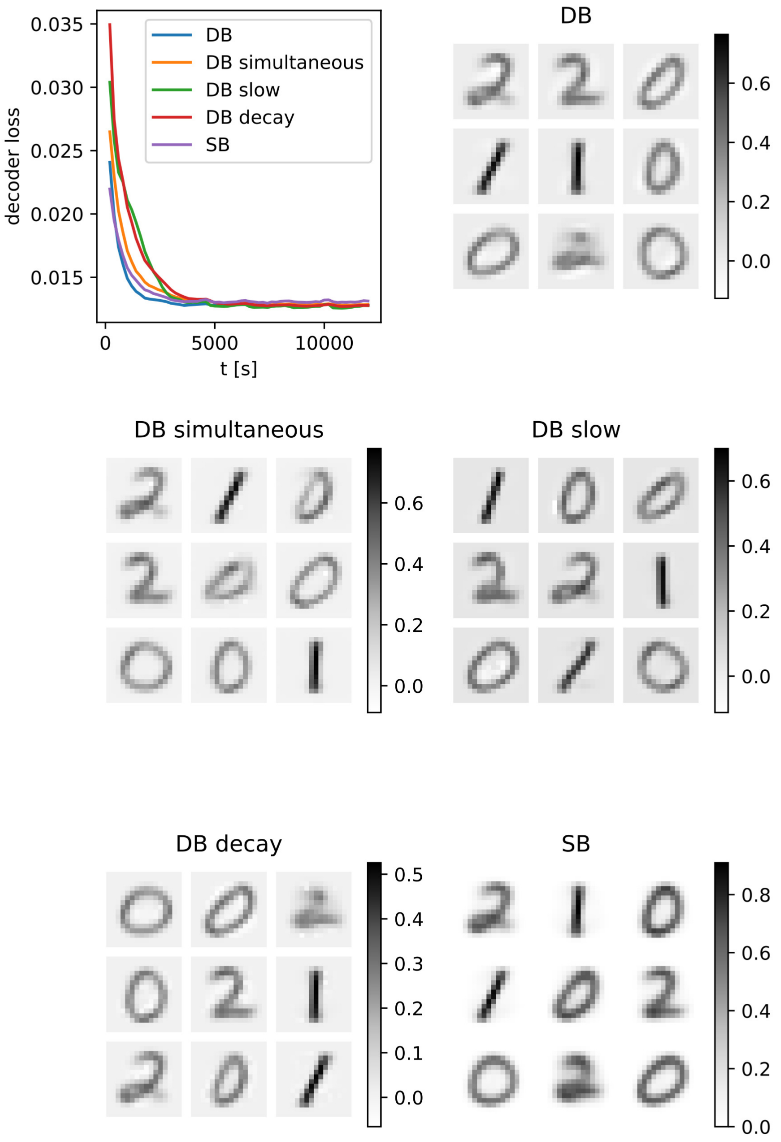

Fig. S2. Comparison of the different learning schemes on the MNIST task. All learning algorithms reach a very similar performance. The dendritic balance learning schemes with slow feedforward adaptation (DB slow) and the weight decay trick (DB decay) converge somewhat slower than dendritic balance with simultaneous feedforward and recurrent adaptation (DB simultaneous), dendritic balance with the analytical solution for recurrent weights (DB) and the somatic balance learning scheme (SB), as expected. DB decay finds smaller weights than other learning schemes, also as expected. As we derived, this can be compensated by a change in the firing threshold, which in our case is done via rapid firing rate adaptation. The learned feedforward weights are also very similar (bottom images).

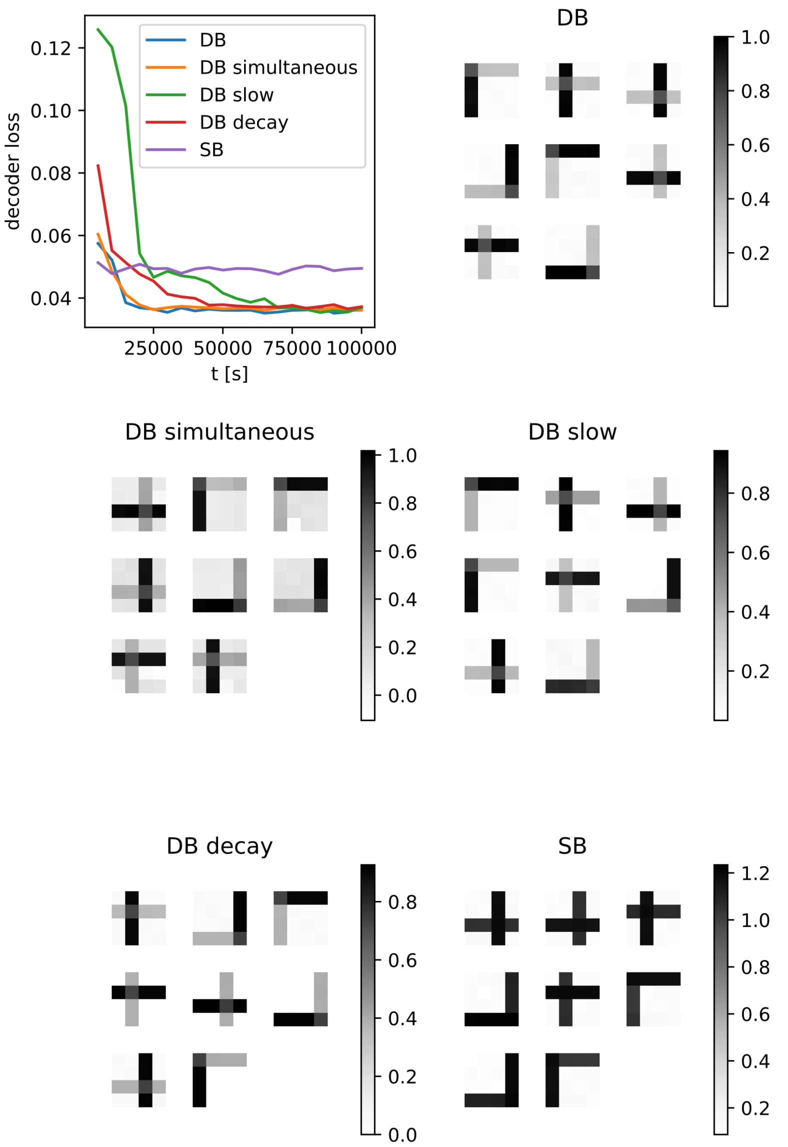

Fig. S3. Comparison of the different learning schemes on the bars task with $p = 0.7$. All dendritic balance algorithms reach a good performance, again DB slow and DB decay converge somewhat later. Learning in the SB network finds a sub-optimal solution. These results are reflected in the learned feedforward weights (bottom), where SB finds representations that do not contain single bars, as it would be optimal, but the collapsed corresponding bars instead.

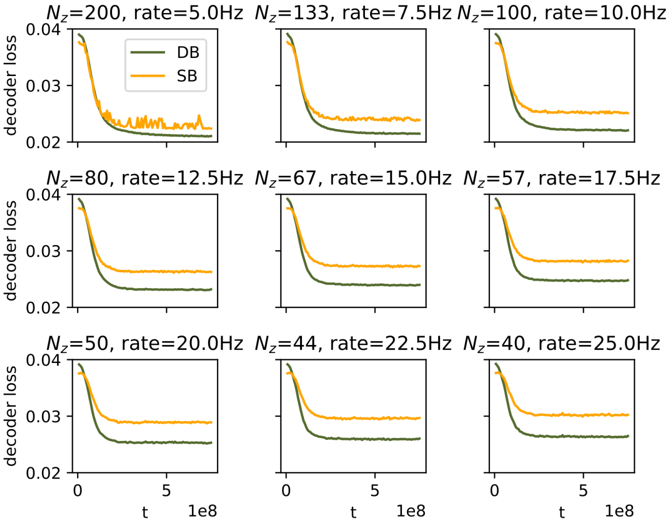

Fig. S4. All learning curves for the natural scenes task (Fig 4).

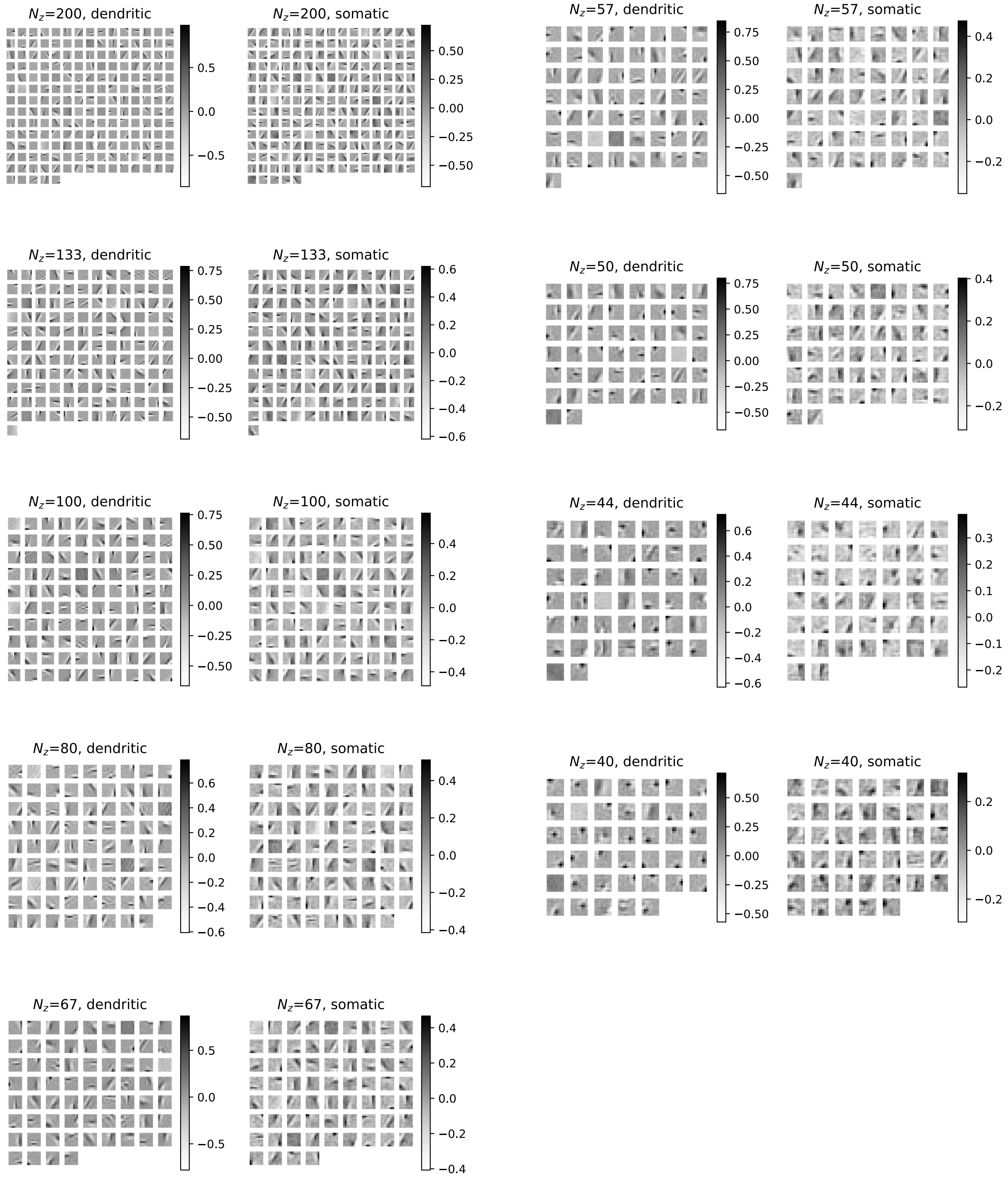

Fig. S5. All learned feedforward weights for the natural scenes task (Fig 4). For a large number of coding neurons neurons in both SB and DB learn weights with Gabor-wavelet like appearance. For smaller networks SB and DB learn qualitatively different weights: DB neurons become detectors for small blobs of activity in the images, similar to center-surround receptive fields. SB neurons also become detectors of blobs of activity but with much less coordination and larger diameter receptive fields.

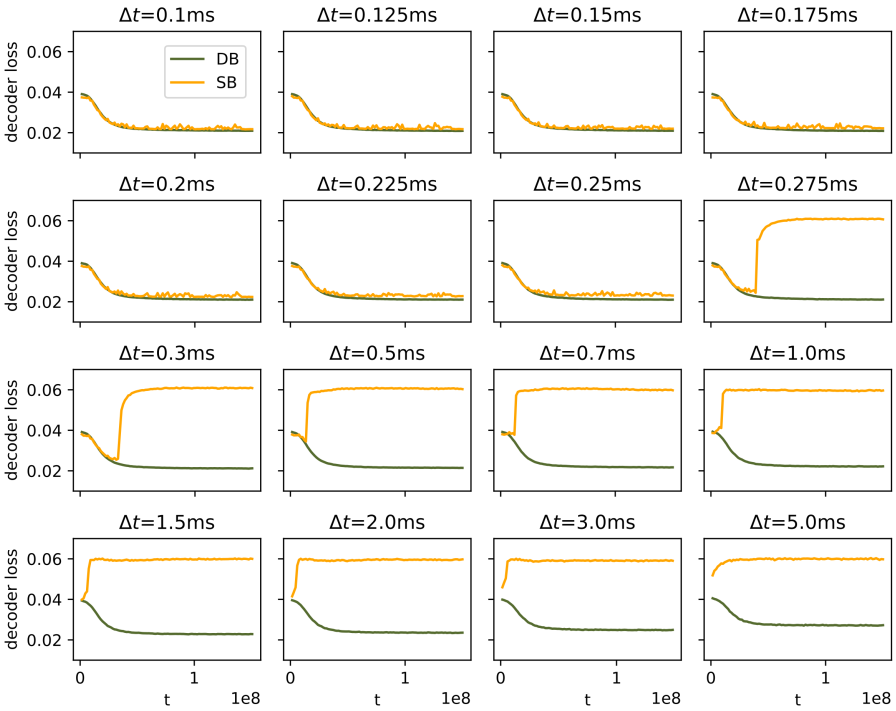

Fig. S6. All learning curves for the natural scenes task for different timesteps (Fig 5A). For long timesteps (i.e. transmission delays) SB learning fails.

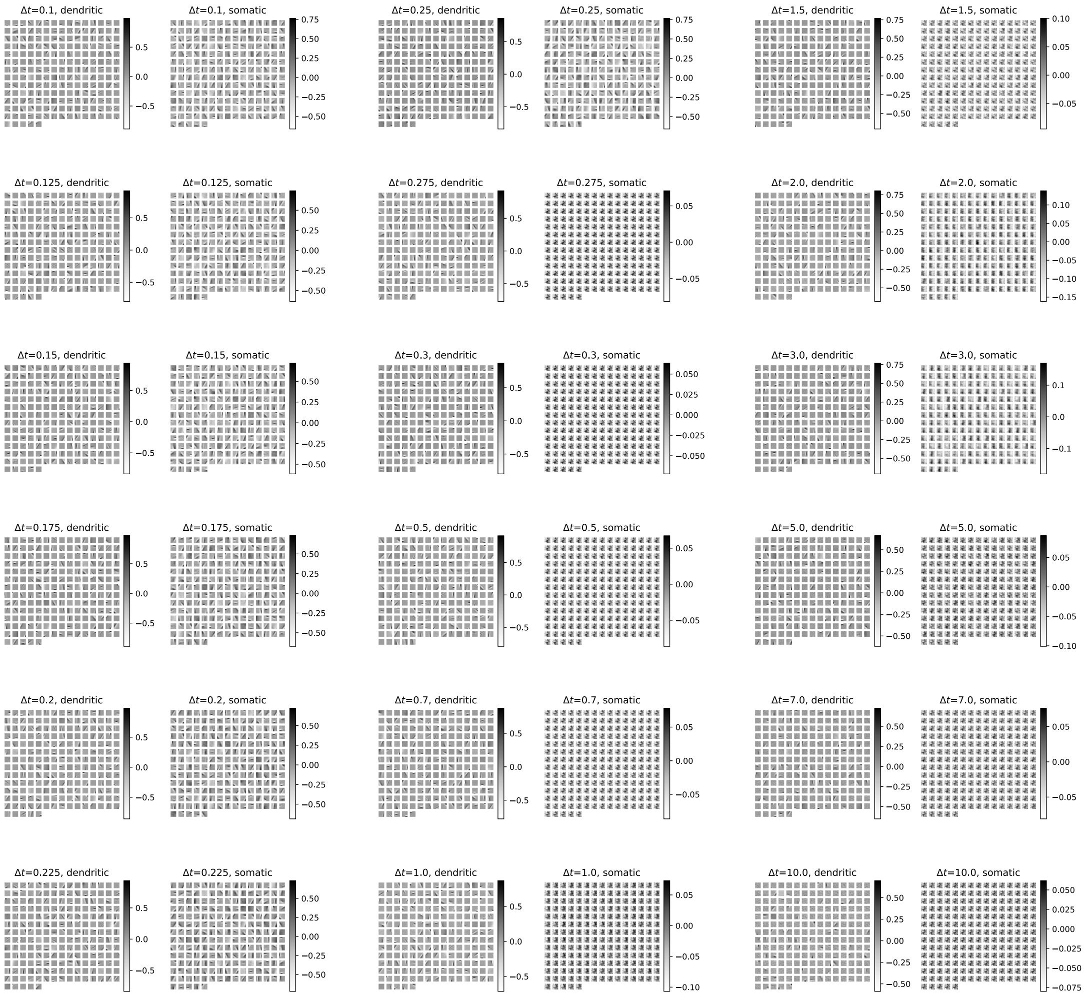

Fig. S7. All learned feedforward weights for the natural scenes task for different timesteps (Fig 5A). For long timesteps the representations learned by SB collapse, while DB continues to find good representations.

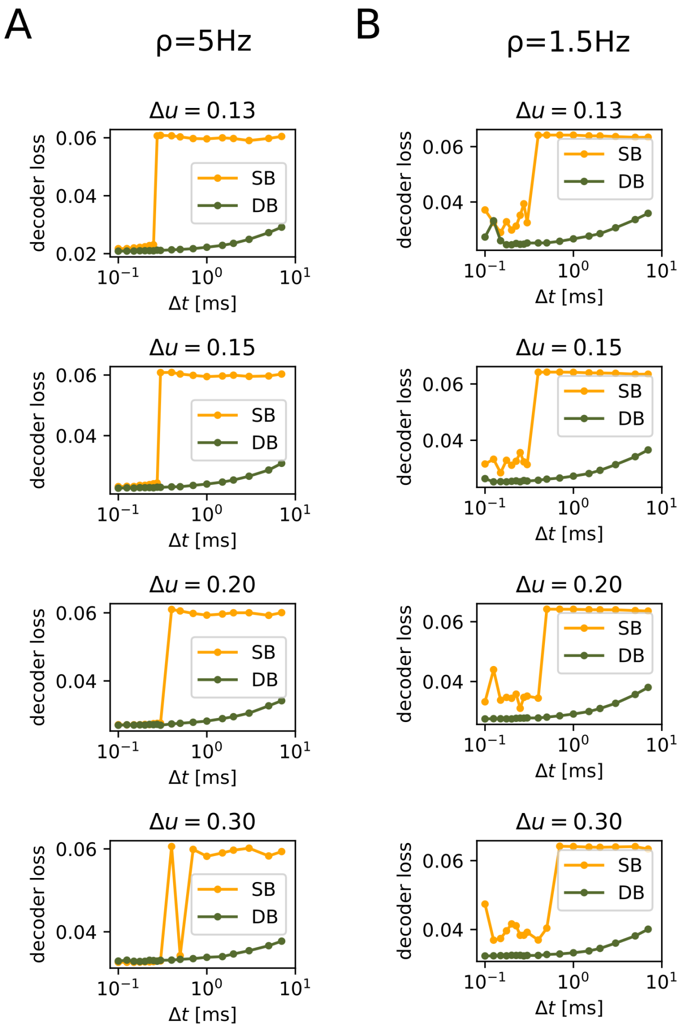

Fig. S8. The results in Fig 5A are robust in respect to the stochasticity of firing Δu and firing rate ρ. We tested firing rates of **A** $\rho = 5$Hz, where learning is mostly stable, and **B** $\rho = 1.5$Hz, where learning becomes slightly unstable. For higher stochasticity (larger Δu) neural firing becomes extremely random, for more deterministic neurons (smaller Δu) learning often does not converge.

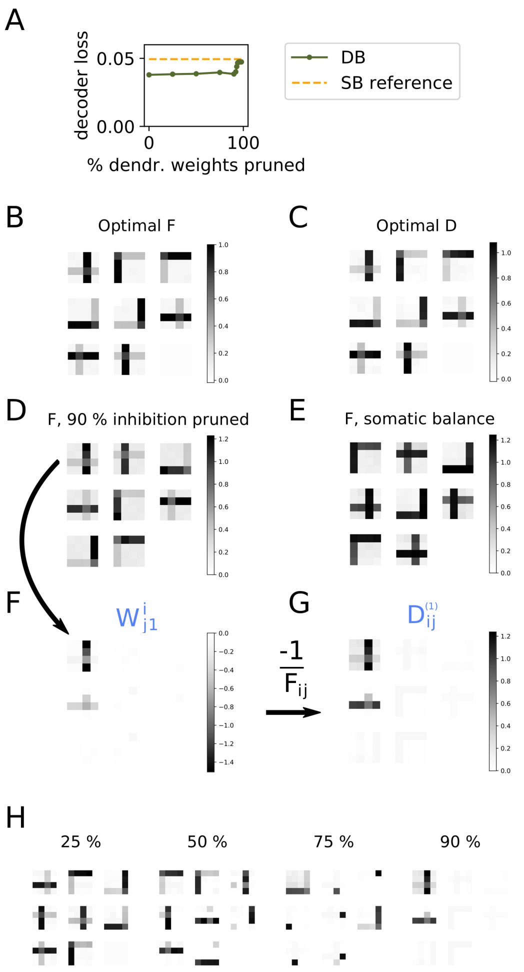

Fig. S9. In the dendritic balance learning scheme many of the dendritic recurrent weights can be pruned while retaining learning performance. We here demonstrate this in the correlated bars task with 4×4 pixel images and $p = 0.6$. The results presented here were obtained with DB with slow feedforward adaptation. Weights were pruned based on the principle presented above. Correlations between neurons were estimated in the first 1% of simulation-time, long before convergence, after which pruning commenced. Several pruned dendritic connections were replaced by a somatic connection to ensure a somatic balance if necessary. **A** 90% of the dendritic recurrent weights can be pruned without losing performance. If more dendritic weights are pruned performance approaches that of the SB learning scheme. **B** Feedforward weights learned with all dendritic connections in place. **C** Decoder corresponding to the network in B. **D** Feedforward weights learned when 90% of dendritic connections are pruned, which are remarkably similar to the optimal solution. **E** In comparison, weights learned by SB find collapsed representations containing two bars, which is a suboptimal solution. **F** Dendritic recurrent weights of neuron 1 in D, after pruning. Weights for a single neuron can be displayed in image space, showing which dendrites they connect to. After pruning with the proposed principle only weights that are important for learning remain. Self-contributions (top left) are always kept and can be computed locally. Other weights only connect to dendrites of the neuron, which codes for the "corresponding" correlated bar (center left). Only this neuron needs the information provided by the recurrent weights for learning, in order to prevent the collapse as we see it in E. **G** Since recurrent plasticity finds a "decoding" by balancing excitation with inhibition, we can find the corresponding "decoder" to the recurrent weights of neuron 1. This decoder only contains the two relevant bars that correspond to one another, demonstrating that our pruning principle can find exactly the relevant contributions and discard all the others. **H** The "decoder" as in G for progressive pruning fractions. It is clearly visible how for larger pruning fractions only the relevant dendritic connections remain.

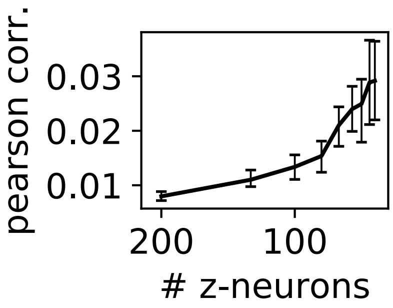

Fig. S10. Average pearson correlation coefficient between outputs $z_i(t)$ of pairs of neurons in DB networks coding for natural images (Fig 4D). The correlation of neural activity increases when the number of neurons decreases. This indicates that the input patterns the neurons learn to represent are more strongly correlated for small networks. Error-bars denote 95% bootstrapping confidence intervals.

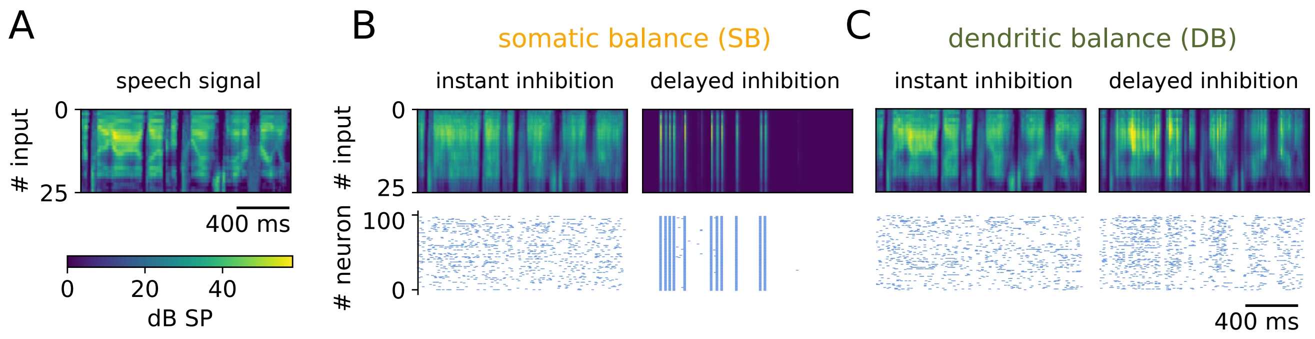

Fig. S11. This figure corresponds to Fig 5F-H, but uses unwhitened input signals. We observed similar results as in Fig 5F-H, here with transmission delays of $\delta = 0.5$ ms. For smaller delays we did not observe a collapse of the population code, likely because whitening leads to stimulus dimensions that have faster temporal dynamics than the original signal, making it harder for inhibition to decorrelate neural responses. For the somatic balance model (SB) the presence of pairwise correlations between inputs requires a different learning rule, where weights are updated according to $\Delta F_{ji} \propto z_j(x_i - \alpha x_i (F\mathbf{x})_j z_j)$ (see (2) for details). Although the dendritic balance model (DB) is also based on a decoder model that assumes inputs with zero pairwise correlations, it still manages to find a very good encoding. **A** Spectrogram of the signal presented in 25 frequency channels. **B** As can be seen in the reconstructed signal (top), SB finds a good encoding for instant inhibition (loss=0.08), but for small delays of 0.5 ms the learned representations collapse, leading to pathological network behavior and bad encoding performance (loss=1.25). **C** In contrast, DB finds a very good encoding for instant inhibition (loss=0.04) and a reasonable encoding with inhibitory delays of 0.5 ms (loss=0.2). Note that whitening changes the scale of the signal, hence decoder losses are not directly comparable between Fig 5 (where the loss is computed on the whitened signal) and Fig S11.
